# HIV infection as vascular risk: A systematic review of the literature and meta-analysis

**DOI:** 10.1371/journal.pone.0176686

**Published:** 2017-05-11

**Authors:** Jose Gutierrez, Ana Letícia A. Albuquerque, Louise Falzon

**Affiliations:** 1 Department of Neurology, Columbia University Medical Center, New York, NY, United States of America; 2 School of Medicine, Federal University of Alagoas, Maceió, AL, Brazil; 3 Center for Behavioral Cardiovascular Health, Columbia University Medical Center, New York, NY, United States of America; China Medical University, TAIWAN

## Abstract

**Importance:**

The vascular risk attributable to HIV infection is rising. The heterogeneity of the samples studied is an obstacle to understanding whether HIV is a vascular risk across geographic regions.

**Objective:**

To test the hypothesis that HIV infection is a vascular risk factor, and that the risk conferred by HIV varies by geographical region.

**Data sources:**

A systematic search of publications was carried out in seven electronic databases: PubMed, The Cochrane Library, EMBASE, Web of Science, LILACS, ClinicalTrials.gov, and WHO International Clinical Trials Registry Platform from inception to July 2015.

**Study selection:**

We included longitudinal studies of HIV+ individuals and their risk of vascular outcomes of ≥ 50 HIV+ cases and excluded studies on biomarkers of vascular disease as well as clinical trials.

**Data extraction and synthesis:**

Data was extracted by one of the authors and independently confirmed by the other two authors. We used incidence rate (IR), incidence risk ratio (IRR) and hazard ratio (HR) with their 95% confidence intervals as measures of risk.

**Main outcome:**

All-death, myocardial infarction (MI), coronary heart disease (CHD), any stroke, ischemic stroke (IS) or intracranial hemorrhage (ICH).

**Results:**

We screened 11,482 references for eligibility, and selected 117 for analysis. Forty-four cohorts represented 334,417 HIV+ individuals, 49% from the United States. Compared with their European counterparts, HIV+ individuals in the United States had higher IR of death (IRR 1.78, 1.69–1.88), MI (IRR 1.61, 1.29–2.01), CHD (IRR 2.27, 1.92–2.68), any stroke (IRR 1.94, 1.59–2.38), IS (IRR 1.56, 1.23–1.98), and ICH (IRR 4.03, 2.72–6.14). Compared with HIV- controls and independent of geographical region, HIV was a risk for death (HR 4.77, 4.55–5.00), MI (HR 1.60, 1.49–1.72), any CHD (HR 1.20, 1.15–1.25), any stroke (HR 1.82, 1.53–2.16), IS (HR 1.27, 1.15–1.39) and ICH (HR 2.20, 1.61–3.02). Use of antiretroviral therapy was a consistent risk for cardiac outcomes, while immunosuppression and unsuppressed viral load were consistent risks for cerebral outcomes.

**Conclusions:**

HIV should be considered a vascular risk, with varying magnitudes across geographical and anatomical regions. We think that strategies to reduce the HIV-related vascular burden are urgent, and should incorporate the disparities noted here.

## Introduction

Vascular disease is the leading cause of global death, [1] and it has become a particular concern in the aging HIV population. The World Health Organization estimates that approximately 37 million people are currently living with HIV/AIDS. [2] Non-infectious comorbidities have emerged as important sources of morbidity and mortality in HIV+ populations, [3] with vascular disease being the leading cause of death among older HIV+ individuals. [4] Whether the rates of vascular disease are a reflection of aging and aging-comorbidities *per se* or HIV-related is an area of ongoing investigation. Furthermore, disparities in rates of vascular disease are influenced by geographical factors that not only reflect greater prevalence of vascular risk factors but also socioeconomic factors that vary regionally.[5] HIV in the United States (US) affects predominantly underserved populations, often living in circumstances that may also influence vascular disease.[6] Additionally, evidence suggests that HIV-related variables such as immunological factors,[7] use of cART, [8] and co-infection with other viruses may modify the risk of vascular disease seen in HIV+ populations.[9] Because the prevalence of these confounders of risk vary depending on the population studied,[10–12] it is plausible that some of disparities in vascular risk seen in HIV+ populations may be attributable to geographical confounders.

Therefore, we conducted a systematic review and a meta-analysis of published medical literature to assess the risk of death and vascular disease among HIV+ individuals to test the hypothesis that HIV infection is a vascular risk factor, and that the risks conferred by HIV varies by geographical region.

## Methods

**Fig 1:**
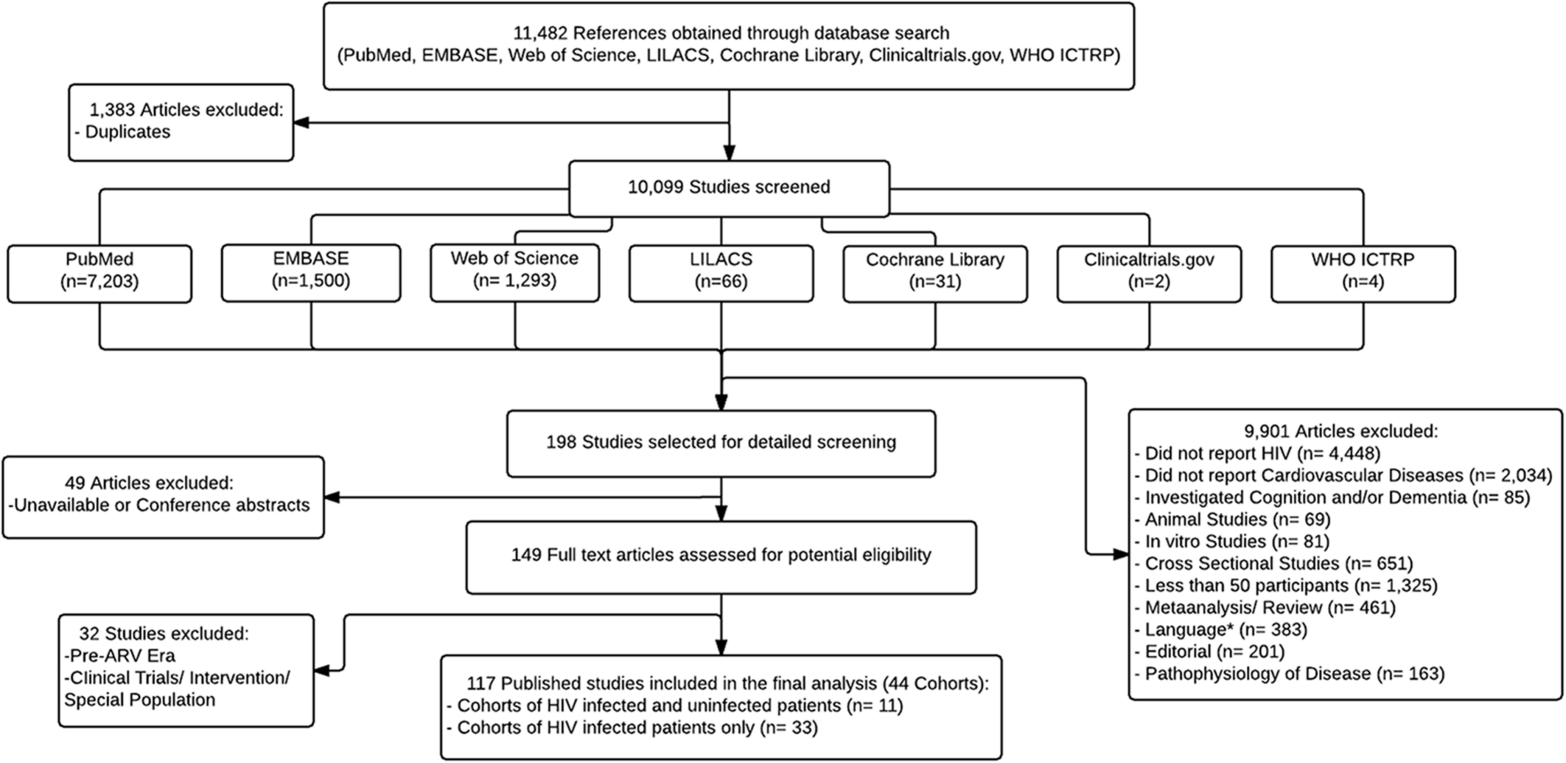
Flow diagram of the systematic search.

Data sources and searches: Our first search was conducted on PubMed on May 26th, 2015 by a librarian (LouF), and 7,203 articles were found. Our second search was carried out in EMBASE, Web of Science and LILACS, within a week (from July 27th to July 31st). 1,172 articles were found to be duplicates from our first search, so we carefully excluded them leaving 2,859 new articles, of which 1,500 were from EMBASE, 1,293 from Web of Science and 66 from LILACS. Our third search was conducted in The Cochrane Library, ClinicalTrials.gov and WHO International Clinical Trials Registry Platform on July 29, 2015, and 248 studies were found. After the exclusion of the non-relevant studies and the duplicates, 37 new articles remained, of which 31 were from The Cochrane Library, 2 from ClinicalTrials.gov and 4 from WHO International Clinical Trials Registry Platform. Therefore, a total of 11,482 studies were screened for inclusion in our meta-analysis (Fig 1, S1 File and S2 File). One publication from a cohort identified through our search was brought to our attention by one of the study investigators.[13].

Longitudinal studies of HIV+ individuals were selected for analysis. The studies were included if they met the following criteria: (1) observational study design; (2) sample size ≥ 50 HIV+ cases, and (3) all-death, myocardial infarction (MI), any coronary heart disease (CHD), any stroke, ischemic stroke (IS) or intracranial hemorrhage (ICH) reported as primary outcome. Studies were excluded if: (1) not about HIV; (2) not about vascular disease; (3) related to cognition and/or dementia; (4) animal studies; (5) in vitro studies; (6) cross-sectional design; (7) small samples (< 50 patients); (8) meta-analysis, systematic reviews and reviews; (9) studies that were not in English, Spanish, French or Portuguese; (10) editorials; (11) studies exploring only pathogenesis; (12) clinical trials (to improve more representative samples of the general population with HIV) and (13) studies from before cART era (<1994).

Study Selection: The first screening in each database was carried out by looking at the title and the abstract of the articles. The second screening consisted of retrieving the full text or extended abstract from pre-screened citations. One of us (AnaA) independently classified the studies as appropriate for further consensus review (between AnaA and JosG) to select studies for this analysis.

Data extraction and quality assurance: We obtained from each of the selected articles the demographic, clinical and laboratory data reported by HIV status. We noted whether the study provided a measure of risk (i.e. Hazard ratios, risk ratio, etc.), and whether the given measure was adjusted for confounders. If the study did not report measures of risks, we calculated crude rate ratios with the provided number of events and sample size. We extracted crude incidence rates when provided. If not provided, we estimated incidence rate and their confidence intervals with the data provided by the authors. If the author did not provide total person-year follow-up, we estimated it by multiplying the follow-up time by the number of subjects and contrasted the estimated incidence rate with the reported in the paper. In the cohorts where HIV negative controls were included, we prioritized obtaining the adjusted hazard ratios, but if not available, we obtained the unadjusted hazard ratio, the risk ratio, or calculated the unadjusted hazard ratio as indicated elsewhere.[14] We used exchangeable incidence rate ratio (IRR), risk ratio, relative risk, with hazard ratios.[15] We noted whether the population study reflected first-time event, recurrent events in populations with known vascular disease, and the geographical region. Finally, we extracted the hazard ratio or risk ratio in cohorts that included HIV only models.

An ideal study would include a random representative sample of the population of HIV in the geographical area of study, compared to a representative or random sample from HIV uninfected controls from the same geographical area. The study must present adjusted hazard ratios by traditional vascular risk factors and ideally would control not only by the presence or absence or vascular risk factors, but also by the degree of control as defined by established clinical guidelines or by using continuous longitudinal measurements of the intensity of such vascular risk factors. A good study would include commodity samples (e.g. administrative datasets, hospital-based, etc.) that included matched (at least by age and sex) HIV- controls, and that reported hazard ratios adjusting for at least demographics and vascular risk factors (expressed categorically or with some continuous measurements measured at baseline). A fair study would report only unadjusted rates of a given outcome.

Data synthesis and Analysis: Demographic, clinical and laboratory data were summarized with simple averages from all the studies that provided them. When a given study provided stratified data, a weighted average was obtained accordingly to be able to harmonize with other studies. Incidence rate and 95% CI (when not provided by the authors) were calculated from raw data using MedCalc Statistical Software version 16.2.1 (MedCalc Software bvba, Ostend, Belgium; https://www.medcalc.org; 2016).

### Statistical analysis

Fixed effects were used for all outcomes. Random effects were used to test robustness of results.[16, 17] The natural logarithm of the HR and their 95% CI for demographic, clinical and laboratory variables were calculated for each study.[14] The average HR and 95% CI for each outcome was obtained using Inverse Variance statistical method provided by Review Manager (RevMan) [Computer program]. Version 5.3. Copenhagen: The Nordic Cochrane Centre, the Cochrane Collaboration, 2014. Heterogeneity was tested only in geographic subgroups and it was quantified with Q test and I^2^. A two-tailed alpha value of ≤ 0.05 was used to determine statistical significance.

## Results

### a) Characteristics of the HIV populations studied

Forty-four cohorts represented 334,417 HIV+ individuals, 49% from the US (Tables A-C in S1 File). The majority of the studies with extractable data and well-defined vascular outcomes came from the US and Europe, which facilitated the comparison between the US and Europe. Using simple linear regression with each study weighted by their sample size, HIV+ individuals in the US had a higher mean age (B = 10.0, P = 0.001), were more likely non-white (B = 27.3, P = 0.005), had viral loads < 1000 copies (B = 5.05, P = 0.001), hepatitis C (B = 24.33, P = 0.01) or higher CD4 counts (B = 48.1, P = 0.01) than their European counterparts. Among the 11 studies that included HIV- controls, HIV+ were more commonly men, non-white, and had a higher prevalence of hypertension, diabetes, dyslipidemia, smoking, hepatitis C, drug use, and baseline cardiac disease (Table D in S1 File).

### b) Incidence rate of death and vascular disease in HIV+ versus HIV- populations (Table 1)

**Table 1:** Incidence rate (per 1,000 person-year) by HIV status and geographical regions.

	All-death(95% CI)	Myocardial infarction(95% CI)	Any Coronary Heart Disease(95% CI)	Any stroke (95% CI)	Ischemic stroke(95% CI)	Intracranial hemorrhage(95% CI)
**HIV +**						
Overall rate	22.42(22.04–22.78)	1.88(1.80–1.95)	4.62(4.51–4.72	1.44(1.32–1.56)	1.08(0.98–1.17)	0.58(0.43–0.72)
United States N = 14 studies	35.15(34.51–35.78)	2.17(2.02–2.32)	4.77(4.66–4.89)	2.97(2.46–3.48)	2.95(2.69–3.22)	1.29(0.92–1.66
Europe N = 13 studies	19.73(18.88–20.57)	1.35(1.24–1.46)	2.10(1.70–2.50)	1.53(1.23–1.83)	1.20(0.94–1.45)	0.32(0.14–0.50)
Mixed low income countries N = 6 studies	49.54(47.44–51.64)	0.60(0.30–1.08	N/A	0.80(0.45–1.15)	N/A	N/A
Mixed high Income countriesN = 8 studies	11.72(11.16–12.28)	3.26(3.07–3.45)	4.85(4.60–5.10)	1.4(1.20–1.50)	0.73(0.62–0.84)	0.83(0.58–1.19)
Recurrent eventsN = 5 studies	60.66(38.48–82.84)	28.16(20.04–36.27)	45.39(33.82–56.82)	6.59(0.01–13.37)	N/A	N/A
**HIV -**						
Overall	4.07(3.92–4.23)	3.00(2.97–3.03)	3.06(3.02–3.11)	2.31(2.21–2.41)	1.05(0.98–1.12	0.22(0.19–0.26)
United States	17.62(17.29–17.96)	3.02(2.99–3.05)	3.23(3.18–3.28)	3.30(2.50–4.30)	2.76(2.61–2.91)	1.05(0.98–1.12)
Europe+ Canada	3.86(3.70–4.01)	2.12(1.89–2.34)	2.40(2.30–2.50)	2.30(2.20–2.40)	0.62(0.54–0.69)	0.17(0.14–0.21)
Recurrent events	23.6(19.8–27.9)	60.3(45.7–78.1)	81.21(64.03–98.39)	N/A	5.3(1.71–12.30)	N/A

Abbreviations: N/A, not available

Among HIV+ individuals, the MI incidence was higher than stroke incidence (IRR 1.31, 1.05–1.63). By defining regions more homogenously, however, HIV+ individuals in the US had a higher stroke incidence than that of MI (IRR 1.37, 1.15–1.64) but less so in Europe (IRR 1.13, 95% CI 0.89–1.44) and in low-to-middle income countries (IRR 1.33, 95% CI 0.94–1.89. Among HIV- individuals in the US, the incidence rates of stroke versus MI were similar (IRR 1.09, 0.93–1.28).

Compared with their European counterparts, HIV+ individuals in the US had a higher IRR for death (1.78, 1.69–1.88), MI (1.61, 1.29–2.01), CHD (2.27, 1.92–2.68), stroke (1.94, 1.59–2.38), IS (2.46, 1.98–3.07), and ICH (4.03, 2.72–6.14). The same geographic disparity was noted among HIV- controls so that those in the US had a higher IRR for death (4.56, 4.08–5.11), MI (1.42, 1.19–1.70), CHD (1.34, 1.14–1.59), stroke (1.43, 1.21–1.71), IS (4.45, 3.37–5.96), and ICH (6.18, 3.68–11.00) than their uninfected European or Canadian counterparts.

### c) Outcomes by HIV status (Tables E-K in S1 File)

#### -Death

**Fig 2:**
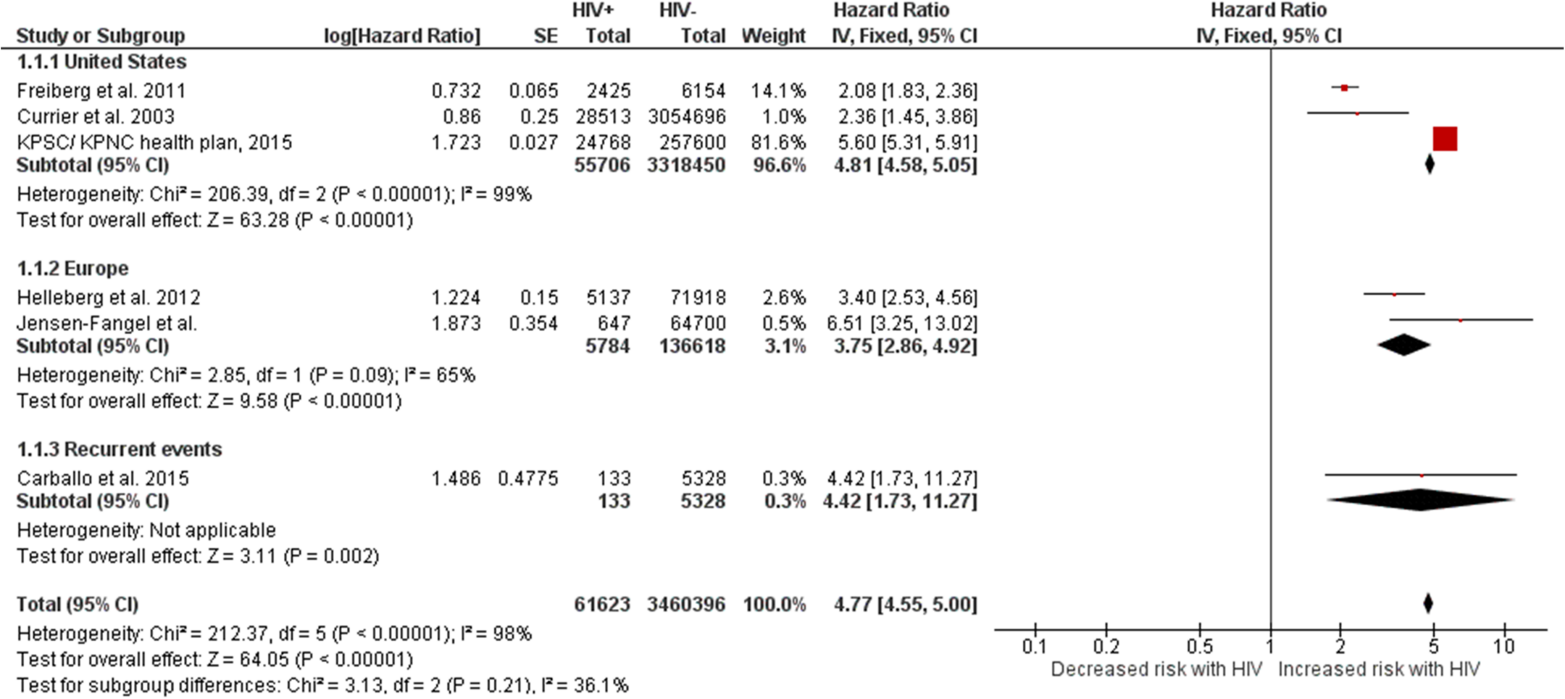
Risk of death in HIV+ individuals vs. HIV- controls. Red squares represent the point estimate for HIV-related vascular risk by study, and the size of each red square represents the weight of each study in the average effect size. Horizontal lines across red squares represent the 95% confidence intervals for the point estimate. The diamonds represent the weighted average point estimate.

HIV infection conferred a four-fold increase in the risk of death compared with HIV- controls (HR 4.77, 4.55–5.00). The point estimate was the highest in the US, but the significance remained similar across geographical regions (Fig 2). Of the six included studies, five did not adjust for confounders; the only study that did adjust for confounders showed a significant 4-fold increase risk of death attributable to HIV.[18] The results were heterogeneous (I^2^ = 98%), but the six individual point estimates were independently significant. Varying the estimation method from fixed effect to random effect did not change the statistical significance but it reduced the point estimate by 23%.

#### -Myocardial infarction

**Fig 3:**
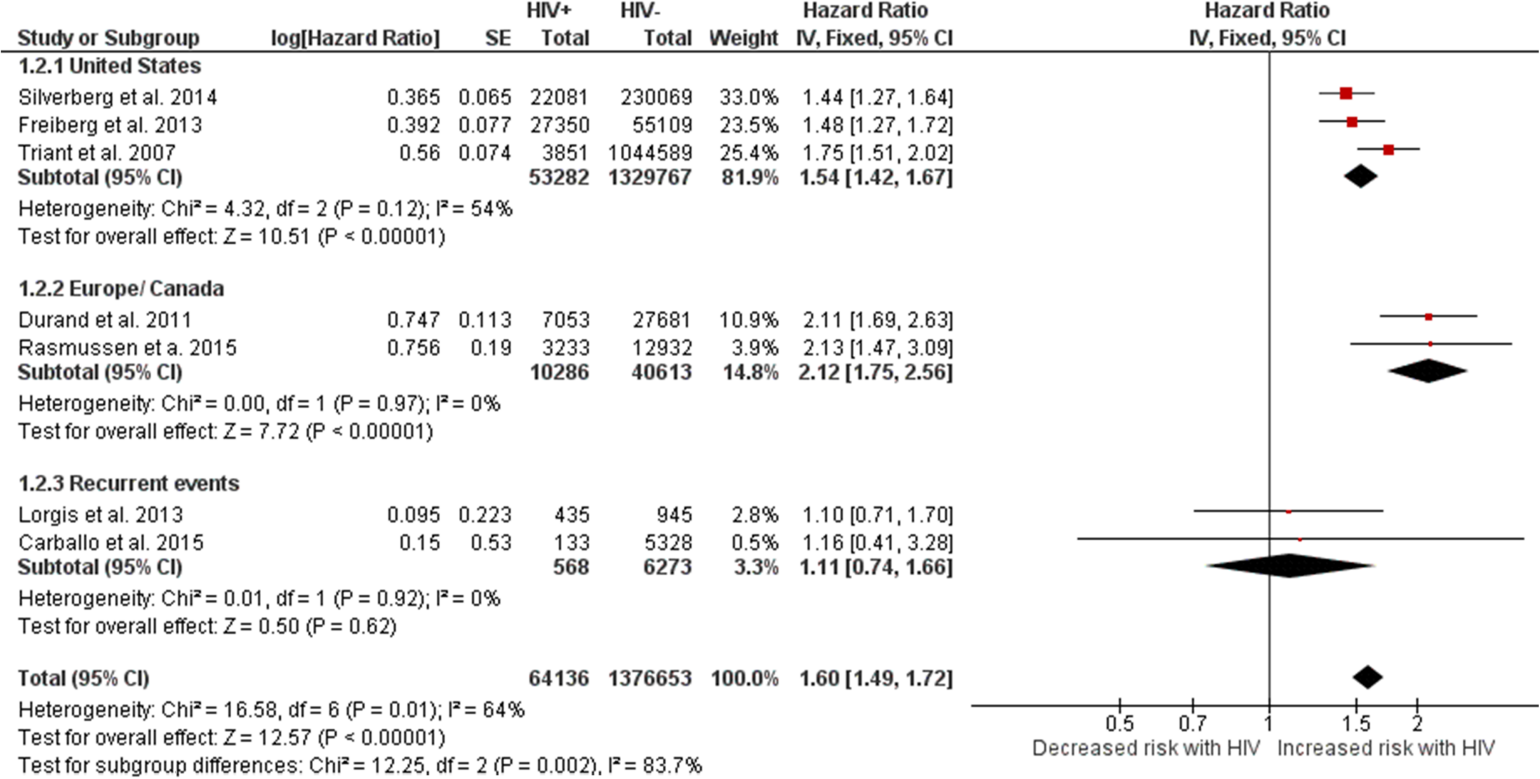
Risk of myocardial infarction in HIV+ individuals vs. HIV- controls. Red squares represent the point estimate for HIV-related vascular risk by study, and the size of each red square represents the weight of each study in the average effect size. Horizontal lines across red squares represent the 95% confidence intervals for the point estimate. The diamonds represent the weighted average point estimate.

HIV+ individuals had a higher risk of MI compared with HIV- controls (HR 1.60, 1.49–1.72, Fig 3). The seven studies included adjusted for at least sex and age, and the studies from administrative datasets in the US and Europe also controlled for vascular risks. The results were significant across regions, but the point estimates were lower for those in the US. The two smallest studies that reported a non-significant increased risk of MI with HIV studied recurrent MI as outcome. The results were moderately heterogeneous (I^2^ = 64%), mostly due to the two studies that showed a point estimate greater than two.[19, 20] The results were insensitive to the effect estimation method.

#### -Any CHD

**Fig 4:**
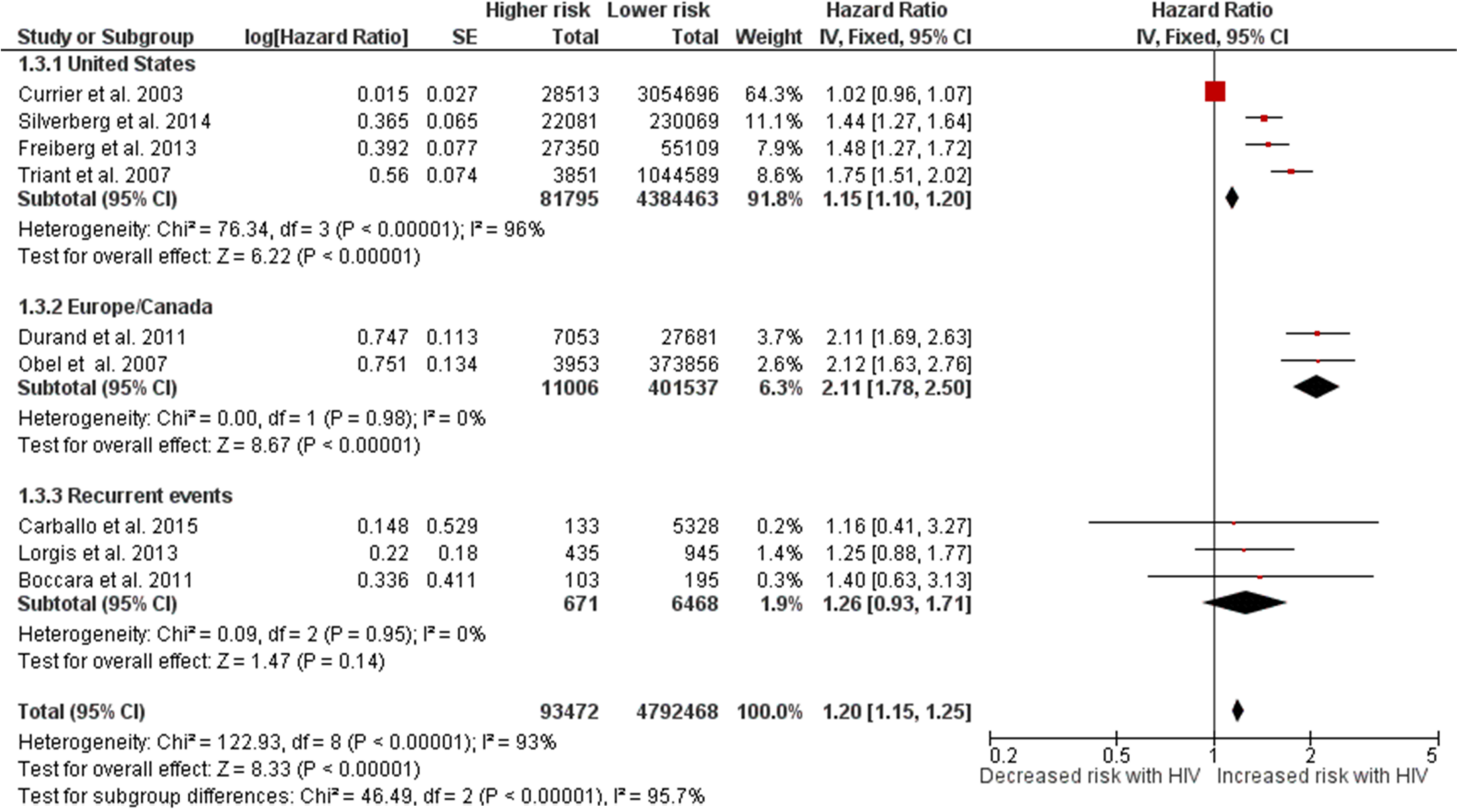
Risk of any coronary artery disease in HIV+ individuals vs. HIV- controls. Red squares represent the point estimate for HIV-related vascular risk by study, and the size of each red square represents the weight of each study in the average effect size. Horizontal lines across red squares represent the 95% confidence intervals for the point estimate. The diamonds represent the weighted average point estimate.

Nine studies had data for any CHD, five referring to MI and 4 to CHD. HIV+ individuals had a higher risk of CHD than HIV- controls (HR 1.20, 1.15–1.25, Fig 4). Taking out the five studies with MI as outcome reduced significantly the strength of the association between HIV and CHD, but it remained significant (HR 1.05, 1.00–1.10). Among the four studies reporting CHD, only one adjusted for multiple confounders, reporting twice the risk of CHD with HIV.[21] The results were heterogeneous as the point estimate ranged from 1.02 to 2.40 (I^2^ = 94%). The results were significant across regions, but the point estimates were lower for those in the US. Furthermore, using a random model rather than fixed model increased the strength of association by 40%.

#### -Any stroke

**Fig 5:**
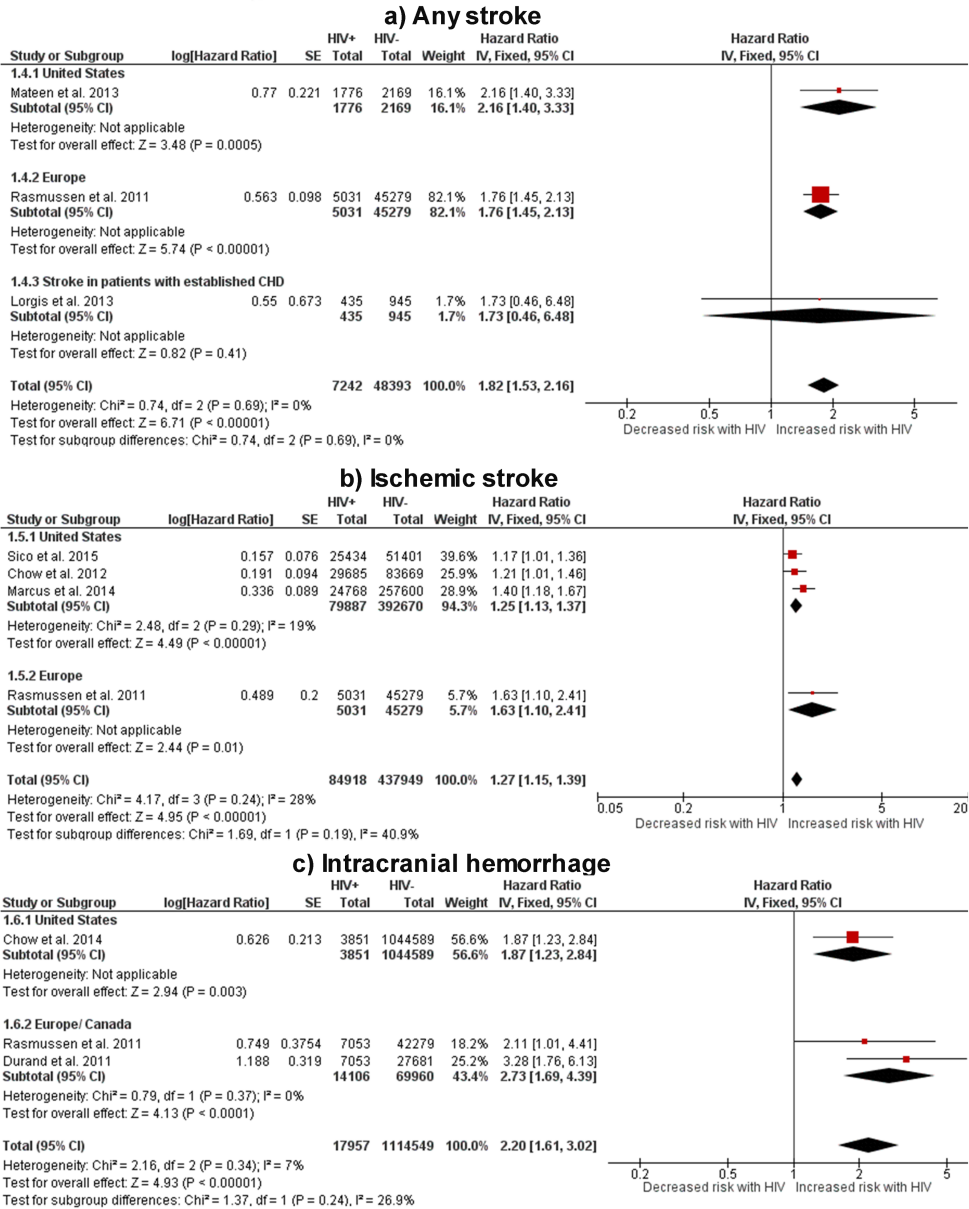
Risk of cerebrovascular events in HIV+ individuals vs. HIV- controls. Red squares represent the point estimate for HIV-related vascular risk by study, and the size of each red square represents the weight of each study in the average effect size. Horizontal lines across red squares represent the 95% confidence intervals for the point estimate. The diamonds represent the weighted average point estimate.

HIV+ individuals had a higher risk of any stroke than HIV- controls (HR 1.82, 1.53–2.16, Fig 5). The results were consistent across the Atlantic, with higher point estimates in the US. The risk of stroke among individuals with established CHD was numerically but not nominally higher among HIV+ individuals. The results were homogenous (I^2^ = 0%) and insensitive to the effect estimation method.

#### -Ischemic stroke

HIV+ individuals had a higher risk of IS than HIV- controls (HR 1.27, 1.15–1.39, Fig 4). The point estimate was higher for the European study included in this comparison, but this study used rate ratio (American studies used HR) and adjusted only for age, sex and country of origin but not for vascular risk factors.[22, 23] The results were homogenous (I2 = 28%) and insensitive to the effect estimation method.

#### -Intracranial hemorrhage

The risk of hemorrhagic stroke was higher in HIV+ individuals compared to uninfected controls (HR 2.20, 1.61–3.02, Fig 4). The risk was adjusted for confounders in all studies. The results were homogenous (I^2^ = 7%) and the point estimate did not change using a random model. The point estimates were higher for the non-American studies than for the American study included in this outcome.

### d) Vascular risks among HIV+ individuals (Tables 2 and 3)

**Table 2:** Meta-analysis of demographic and vascular risk among HIV infected cohorts.

Outcome	Age (per 5-year)	Male sex	Non-white ethnicity	Dyslipidemia	HTN	DM	Smoking
**All-death**	**1.10,1.05–1.15**	**1.27,1.14–1.42**	**0.81,0.47–1.40**	**0.91,0.84–0.98(per 10 mg/dl chol)**	**0.98,0.85–1.14**	**1.45,1.22–1.71**	**1.13,0.97–1.31**
**# studies**	**4 studies**	**7 studies**	**2 studies**	**1 study**	**1 study**	**1 study**	**3 studies**
**Myocardial infarction**	**1.25,1.22–1.28**	**1.97,1.51–2.57**	**0.79,0.72–0.88**	**2.21,1.85–2.65**	**1.84,1.60–2.12**	**1.53,1.32–1.76**	**2.21,1.94–2.51**
**# studies**	**4 studies**	**5 studies**	**3 studies**	**4 studies**	**6 studies**	**6 studies**	**6 studies**
**Any Coronary Heart Disease**	**1.41,1.36–1.47**	**1.76,1.33–2.32**	**0.61,0.17–2.17**	**2.88,2.55–3.25**	**4.60,4.06–5.21**	**1.73,1.55–1.92**	**1.62,1.23–2.12**
**# studies**	**2 studies**	**1 study**	**1 study**	**1 study**	**1 study**	**2 studies**	**1 study**
**Any stroke**	**1.10,1.05–1.15**	**1.11,0.83–1.49**	**0.89,0.46–1.74**	**3.02,1.48–6.17**	**1.67,1.47–1.90**	**1.32,0.94–1.85**	**1.35,1.11–1.65**
**# studies**	**4 studies**	**2 studies**	**1 study**	**1 study**	**4 studies**	**2 studies**	**3 studies**
**Ischemic stroke**	**1.34,1.16–1.54**	**1.03,0.53–2.00**	**0.71,0.41–1.25**	**0.99,0.55–1.80**	**0.79,0.44–1.45**	**0.59,0.28–1.23**	**0.83,0.49–1.40**
**# studies**	**1 study**	**1 study**	**1 study**	**1 study**	**1 study**	**1 study**	**1 study**
**Intracranial hemorrhage**	N/A	**0.56,0.24–1.28**	N/A	N/A	N/A	*	N/A
**# studies**		**1 study**					

Abbreviations: N/A, not available

**Table 3:** Meta-analysis of HIV-related variables and coinfections among HIV infected cohorts.

Outcome	cART	CD4 count	Viral load	Drug Use	HBV	HCV
**All-death**	**5.60,3.18–9.86(cART naive)**	**2.98.2.67–3.32(<200 cells/ul)**	**1.65,1.50–1.81(>10-100K)**	**1.74,1.50–1.81**	N/A	**1.74,1.55–1.96**
**# studies**	**1 study**	**5 studies**	**4 studies**	**5 studies**	**-**	**2 studies**
**Myocardial infarction**	**Any cART: 1.20, 1.09–1.33Abacavir (any): 1.77, 1.50–2.10PI (per year): 1.09, 1.08–1.47)**	**1.01,0.78–1.32 (<200 cells/ul)**	**1.04,0.98–1.09(per log**^**10**^**)**	**0.95,0.66–1.37**	**0.96,0.75–1.21**	**1.09,0.89–1.33**
**# studies**	**3,3, and 2 studies, respectively.**	**2 study**	**2 studies**	**2 studies**	**1 study**	**2 studies**
**Any Coronary Heart Disease**	**Any cART: 1.05, 0.57–1.43Abacavir (any): 1.66, 1.41–1.94Tenofovoir (any): 0.91, 0.57–1.43)**	**1.02, 0.93–1.12(Per 50 cell)**	**0.93,0.54–1.61(HIV RNA > 400)**	N/A	**0.77,0.99–5.88**	**3.94,1.00–15.5**
**# studies**	**3, 2, and 2 studies, respectively.**	**1 study**	**2 studies**		**1 study**	**1 study**
**Any stroke**	**Any cART: 4.16, 0.80–21.65Abacavir: 2.10, 1.20–3.66**	**1.91,1.44–2.54 (CD4 < 200)**	**3.97, 1.90–8.31 (HIV RNA > 400)**	N/A	N/A	**1.22,1.06–1.40**
**# studies**	**1 study for each comparison**	**3 studies**	**1 study**	**-**	**-**	**2 studies**
**Ischemic stroke**	**NRTI use: 1.19 (0.51–2.79)NNRTI use: 0.38 (0.19–0.76)PI use: 0.63 (0.30–1.33)**	**0.97,0.90–1.05 (CD4 nadir per 50)**	**1.10 (1.04–1.17) per log**	N/A	N/A	N/A
**# studies**	**1 study**	**1 study**	**1 study**			
**Intracranial hemorrhage**	**0.46,0.18–1.15**	**4.61, 2.09–10.2 (< 200)**	N/A	N/A	N/A	N/A
**# studies**	**1 study**	**1 study**				

Abbreviations: N/A, not available.

Older age was the more consistent risks for outcomes among HIV+ individuals. While male sex was a significant predictor of MI (HR 1.97, 1.51–2.57) and CHD (HR 1.76, 1.33–2.32); it was not for stroke (HR 1.11, 0.83–1.49). Traditional vascular risks predicted MI/CHD and stroke, but not IS. The study used to extract HR for IS used atrial fibrillation, structural heart disease and anticoagulant/antiplatelet together with traditional vascular risks,[23] which confounds the role of risk factors into a given stroke etiology.

While the risk of death was higher among cART naïve patients (HR 5.60, 3.18–9.86), the risk of MI (HR 1.20, 1.09–1.33) was higher with the use of cART, especially abacavir (HR 1.77, 1.50–2.10) and protease inhibitors (HR 1.09 per year of use, 1.08–1.47). While abacavir was also identified as a risk factor for stroke (HR 2.10, 1.20–3.66), the use of cART or different subtypes of cART was less consistently reported as a risk factor for IS or ICH. While the role of CD4 counts was equivocal for MI/CHD, CD4 counts < 200 cells was a risk factor for death (HR 2.98, 2.67–3.32), stroke (HR 1.91, 1.44–2.54) and ICH (HR 4.61, 2.09–10.2). Similarly, the risk of MI/CHD due to unsuppressed viral replication is equivocal, while unsuppressed viral replication is a risk for death (HR 1.65 if >10-100K, 1.50–1.81), stroke (HR 3.97 if > 400 copies, 1.90–8.31), and IS (HR 1.10 per log, 1.04–1.17). While the use of drug use and hepatitis b were less consistently related with vascular outcomes, Hepatitis C emerged as a consistent risk for death (HR 1.74, 1.55–1.96), CHD (HR 3.94, 1.00–15.5) and stroke (HR 1.22, 1.06–1.40).

## Discussion

The cumulative evidence presented here suggests that HIV+ individuals have a higher risk of death and vascular disease than uninfected populations. The results were consistent even in studies that used adjusted models of good quality and in those that used referent uninfected populations with similar socioeconomic background.[12, 24, 25] Although there exist unmeasured variability in vascular risk factor control and other possible confounders, this deficit in methodology does not change the public health implication that HIV+ populations are at a greater risk of vascular disease than uninfected controls. Furthermore, the increased vascular risk is consistent across geographical regions, which adds strength to our main results. The magnitude of the risk, however, differs by region, with greater rates of vascular disease noted in HIV+ individuals in the US compared to those living in Europe. Similar geographic disparities were also noted for HIV- controls living in the US compared to those in Europe, and this disparity is not exclusive to the HIV- controls included in this meta-analysis, but also noted in studies more representative of the general population in US and Europe.[26, 27] Together, these data suggest that individuals in the US included in this meta-analysis (predominantly from matched underserved populations) and in general have poorer vascular health than those in Europe. The infection with HIV accentuates even further such disparity.

The disparities in risk attributed to HIV may have several explanations. First, the incidence rates used in our analysis are averages of several years. As the care for those with HIV has improved, the rates of vascular risk have decreased and the relative risk compared with uninfected controls has attenuated. [13, 28–30] Also, because greater risk of vascular events is often noted after initiation of cART in patients with low CD4 counts compared to starting it when CD4 counts are higher, [21, 31] more recent event rates may be lower as greater emphasis is given to start cART earlier in the course of the infection.[32] The higher prevalence of vascular risk factors among HIV+ individuals included in this study (Table D in S1 File) or HIV+ individuals in the US (Table L in S1 File) compared to uninfected referent populations may be another contributor to the disparity in vascular risks. Traditional vascular risks confer a higher risk of death and vascular disease (Table 3), and thus represent a clear target for intervention. It is a major limitation that several studies focused on vascular disease did not report rates of vascular risk factors, and that only a minority of studies reported the degree of control of such risks, so an even greater proportion of the HIV-related disparity in vascular risk attributable to traditional vascular risk factors cannot be excluded. Arterial inflammation may also play a role in HIV-related vascular disease. For example, evidence exists of arterial inflammation in the aorta and brain arteries accompanied by activation of soluble blood biomarkers of inflammation, which relates to biomarkers of vascular risk.[33, 34] Accelerated atherosclerosis has been documented in HIV+ individuals as documented by a higher carotid intima-media thickness and coronary artery calcium rate of progression mostly driven by traditional vascular risk factors, with a lesser role for higher viral loads and CD4 nadir < 200.[35, 36] Determining the triggers for arterial inflammation may offer new therapeutic venues in this population.

Coronary heart disease is predominantly due to atherosclerosis, and the risk is typically related to traditional vascular risk factors and cART, especially abacavir (Table 3).[37] Stroke during HIV infection, on the other hand, is a heterogeneous disease where traditional vascular risk factors play only a partial role. For example, the Framingham risk score is a better predictor of biomarkers for CHD [38] than for stroke risk among HIV^+^ individuals.[39] Studies that define stroke more broadly do reproduce known associations between stroke and vascular risk factors among those with HIV, but reports that disclose the mechanisms of stroke in more details provide etiologies not typically found in cardiac disease, such as hypercoagulable state, vasculitis, and HIV vasculopathy.[40–43] These data suggest the need for greater discrimination in mechanistic studies between cerebral and cardiac outcomes, so that targeted preventive measures can be appropriately applied.

Results of this study should be interpreted in light of the following limitations. The populations studied are heterogeneous, most outcomes are obtained from administrative datasets, and adjudication of vascular events was usually not confirmed by chart review. The studies we included over-represent HIV+ populations on cART and results may not apply to the entire HIV+ population. However, given that HIV suppression, immune competence, and access to care protect against death and vascular events, vascular risks of HIV may be even greater for these underrepresented in this study. This bias is not a minor issue: in the US alone, more than 1.2 million people live with HIV and over 50,000 new cases of HIV infection are diagnosed each year.[44] Of this population, 14% are unaware of their diagnosis, 60% do not engage in care, and only a minority received cART or achieve viral suppression, with the lowest rate of viral suppression noted in the youngest cohort. Because one out of four newly HIV-infected individuals and over 100,000 currently living with HIV are between 13–24 years old, [45] studying HIV-related vascular disease represents a major public health challenge due to high indirect costs of vascular disease in the young such as longer years of lost productivity and premature death attributable to vascular disease.

## Supporting information

S1 File **Table A:** Demographic, clinical and immunological characteristics of HIV cohorts studied at baseline enrollment **Table B: **General characteristics of the studies including HIV+ cases compared with HIV- controls **Table C:** Weighted averages of study characteristics of the HIV samples studied across geographical regions **Table D:** Difference in demographic and clinical characteristics in HIV+ compared with HIV- cases **Table E:** All-death risks among HIV+ samples **Table F:** Myocardial infarction risks among HIV+ individuals **Table G:** Coronary Heart Disease risks among HIV+ individuals **Table H:** Cerebrovascular risks among HIV+ individuals **Table I:** Vascular death risks among HIV+ individuals **Table J:** Any vascular disease risks among HIV+ individuals **Table K:** Other Vascular Outcomes risks among HIV+ individuals **Table L:** Characteristics of the HIV studies from the US included in this study and the NHANES-derived characteristics of HIV infected individuals in the United States and the general uninfected population.
(PDF)

S2 File PRISMA statement. (PDF)
