# Supplementary material for: HIV infection as vascular risk: A systematic review of the literature and meta-analysis
Source: PLoS One. 2017 May 11;12(5):e0176686. doi: 10.1371/journal.pone.0176686 (PMC5426615; doi:10.1371/journal.pone.0176686)
Supplement: S1 File — Table A: Demographic, clinical and immunological characteristics of HIV cohorts studied at baseline enrollment Table B: General characteristics of the studies including HIV+ cases compared with HIV- controls Table C: Weighted averages of study characteristics of the HIV samples studied across geographical regions Table D: Difference in demographic and clinical characteristics in HIV+ compared with HIV- cases Table E: All-death risks among HIV+ samples Table F: Myocardial infarction risks among HIV+ individuals Table G: Coronary Heart Disease risks among HIV+ individuals Table H: Cerebrovascular risks among HIV+ individuals Table I: Vascular death risks among HIV+ individuals Table J: Any vascular disease risks among HIV+ individuals Table K: Other Vascular Outcomes risks among HIV+ individuals Table L: Characteristics of the HIV studies from the US included in this study and the NHANES-derived characteristics of HIV infected individuals in the United States and the general uninfected population. (PDF) [file pone.0176686.s001.pdf]

HIV INFECTION AS VASCULAR RISK: A SYSTEMATIC REVIEW OF THE LITERATURE AND META-ANALYSIS.

SUPPORTING INFORMATION TABLE OF CONTENT

	Page
**1.-Supporting methods**	**2**
**1.1: Data search strategy**	**2**
**1.2: Data search detailed methods & collection process**	**5**
**1.3: Data characterization**	**6**
**2.-Supporting results**	**7**
**Table A: Demographic, clinical and immunological characteristics of HIV cohorts studied at baseline enrollment**	**7**
**Table B: General characteristics of the studies including HIV+ cases compared with HIV- controls**	**12**
**Table C: Weighted averages of study characteristics of the HIV samples studied across geographical regions**	**14**
**Table D: Difference in demographic and clinical characteristics in HIV+ compared with HIV- cases**	**14**
**Table E: All-death risks among HIV+ samples**	**15**
**Table F: Myocardial infarction risks among HIV+ individuals**	**16**
**Table G: Coronary Heart Disease risks among HIV+ individuals**	**17**
**Table H: Cerebrovascular risks among HIV+ individuals**	**18**
**Table I: Vascular death risks among HIV+ individuals**	**19**
**Table J: Any vascular disease risks among HIV+ individuals**	**20**
**Table K: Other Vascular Outcomes risks among HIV+ individuals**	**21**
**Table L: Characteristics of the HIV studies from the US included in this study and the NHANES-derived characteristics of HIV infected individuals in the United States and the general uninfected population.**	**22**
**4.-References**	**24**

1- SUPPORTING METHODS

1.1: Data search strategy:

1.1.1: PubMed: Searched from inception to 25/5/2015

(("hiv"[MeSH Terms] OR "hiv"[All Fields]) OR ("acquired immunodeficiency syndrome"[MeSH Terms] OR ("acquired"[All Fields] AND "immunodeficiency"[All Fields] AND "syndrome"[All Fields]) OR "acquired immunodeficiency syndrome"[All Fields] OR "aids"[All Fields]) OR ("hiv"[MeSH Terms] OR "hiv"[All Fields] OR ("human"[All Fields] AND "immunodeficiency"[All Fields] AND "virus"[All Fields]) OR "human immunodeficiency virus"[All Fields])) AND (("stroke"[MeSH Terms] OR "stroke"[All Fields]) OR lacunar[All Fields] OR ("intracranial haemorrhage"[All Fields] OR "intracranial hemorrhages"[MeSH Terms] OR ("intracranial"[All Fields] AND "hemorrhages"[All Fields]) OR "intracranial hemorrhages"[All Fields] OR ("intracranial"[All Fields] AND "hemorrhage"[All Fields]) OR "intracranial hemorrhage"[All Fields]) OR ("atrial fibrillation"[MeSH Terms] OR ("atrial"[All Fields] AND "fibrillation"[All Fields]) OR "atrial fibrillation"[All Fields]) OR ("vascular diseases"[MeSH Terms] OR ("vascular"[All Fields] AND "diseases"[All Fields]) OR "vascular diseases"[All Fields] OR ("vascular"[All Fields] AND "disease"[All Fields]) OR "vascular disease"[All Fields]) OR ("myocardial infarction"[MeSH Terms] OR ("myocardial"[All Fields] AND "infarction"[All Fields]) OR "myocardial infarction"[All Fields]) OR ("myocardial infarction"[MeSH Terms] OR ("myocardial"[All Fields] AND "infarction"[All Fields]) OR "myocardial infarction"[All Fields] OR ("heart"[All Fields] AND "attack"[All Fields]) OR "heart attack"[All Fields]) OR revascularization[All Fields] OR ("stents"[MeSH Terms] OR "stents"[All Fields] OR "stenting"[All Fields]) OR ("carotid artery diseases"[MeSH Terms] OR ("carotid"[All Fields] AND "artery"[All Fields] AND "diseases"[All Fields]) OR "carotid artery diseases"[All Fields] OR ("carotid"[All Fields] AND "atherosclerosis"[All Fields]) OR "carotid atherosclerosis"[All Fields]) OR ("heart failure"[MeSH Terms] OR ("heart"[All Fields] AND "failure"[All Fields]) OR "heart failure"[All Fields] OR ("congestive"[All Fields] AND "heart"[All Fields] AND "failure"[All Fields]) OR "congestive heart failure"[All Fields]) OR ("peripheral arterial disease"[MeSH Terms] OR ("peripheral"[All Fields] AND "arterial"[All Fields] AND "disease"[All Fields]) OR "peripheral arterial disease"[All Fields] OR ("peripheral"[All Fields] AND "artery"[All Fields] AND "disease"[All Fields]) OR "peripheral artery disease"[All Fields]) OR ("aortic aneurysm"[MeSH Terms] OR ("aortic"[All Fields] AND "aneurysm"[All Fields]) OR "aortic aneurysm"[All Fields]) OR (("aorta"[MeSH Terms] OR "aorta"[All Fields] OR "aortic"[All Fields]) AND ("atherosclerosis"[MeSH Terms] OR "atherosclerosis"[All Fields])))) AND "humans"[MeSH Terms]

1.1.2: The Cochrane Library: All databases searched 7/29/2015

#1 MeSH descriptor: [HIV] explode all trees
#2 hiv:ti,ab
#3 "human immunodeficiency virus":ti,ab
#4 MeSH descriptor: [Acquired Immunodeficiency Syndrome] explode all trees
#5 "acquired immune deficiency syndrome":ti,ab
#6 aids:ti,ab
#7 #1 or #2 or #3 or #4 or #5 or #6
#8 MeSH descriptor: [Stroke] explode all trees
#9 stroke:ti,ab
#10 lacunar:ti,ab
#11 "intracranial haemorrhag*":ti,ab

#12 MeSH descriptor: [Atrial Fibrillation] explode all trees
#13 "atrial fibrillation":ti,ab
#14 MeSH descriptor: [Vascular Diseases] explode all trees
#15 "vascular disease*" ti,ab
#16 MeSH descriptor: [Myocardial Infarction] explode all trees
#17 ("heart infarction*" or "heart attack*" or "myocardial infarction*"):ti,ab
#18 revascularization:ti,ab
#19 MeSH descriptor: [Stents] explode all trees
#20 MeSH descriptor: [Stents] explode all trees
#21 stent*:ti,ab
#22 MeSH descriptor: [Carotid Artery Diseases] explode all trees
#23 "carotid artery disease*":ti,ab
#24 "carotid atherosclerosis":ti,ab
#25 MeSH descriptor: [Heart Failure] explode all trees
#26 "heart failure":ti,ab
#27 MeSH descriptor: [Peripheral Arterial Disease] this term only
#28 "peripheral occlusive artery disease":ti,ab
#29 aorta:ti,ab
#30 MeSH descriptor: [Aortic Aneurysm] explode all trees
#31 "aortic aneurysm":ti,ab
#32 MeSH descriptor: [Atherosclerosis] explode all trees
#33 atherosclerosis:ti,ab
#34 #8 or #9 or #10 or #11 or #12 or #13 or #14 or #15 or #16 or #17 or #18 or #19 or #20 or #21 or #22 or #23 or #24 or #25 or #26 or #27 or #28 or #29 or #30 or #31 or #32 or #33
#35 #7 and #34

<u>1.1.3: EMBASE:</u> Searched from 1980 to 29 Jul 2015

#41. #7 AND #32 AND #38 AND [humans]/lim AND [embase]/lim
#38. #33 OR #34 OR #35 OR #36 OR #37
#37. cohort*:ab,ti
#36. 'follow up'/de
#35. 'prospective study'/de
#34. 'longitudinal study'/exp
#33. 'cohort analysis'/de
#32. #8 OR #9 OR #10 OR #11 OR #12 OR #13 OR #14 OR #15 OR #16 OR #17 OR #18 OR #19 OR #20 OR #21 OR #22 OR #23 OR #24 OR #25 OR #26 OR #27 OR #28 OR #29 OR #30 OR #31
#31. atherosclerosis:ab,ti

#30. 'atherosclerosis'/exp
#29. 'aortic aneurysm':ab,ti
#28. 'aorta':ab,ti
#27. 'peripheral occlusive artery disease'/exp
#26. 'heart failure':ab,ti
#25. 'heart failure'/exp
#24. 'carotid atherosclerosis':ab,ti
#23. 'carotid artery diseases':ab,ti
#22. 'carotid artery disease'/exp
#21. stents:ab,ti OR stenting:ab,ti
#20. 'stent'/exp
#19. revascularization:ab,ti
#18. 'heart attack':ab,ti
#17. 'myocardial infarction':ab,ti
#16. 'heart infarction'/exp
#15. 'vascular disease':ab,ti OR 'vascular diseases':ab,ti
#14. 'vascular disease'/exp
#13. 'atrial fibrillation':ab,ti
#12. 'atrial fibrillation'/exp
#11. 'intracranial haemorrhage':ab,ti OR 'intracranial haemorrhages':ab,ti
#10. lacunar:ab,ti
#9. stroke:ab,ti
#8. 'cerebrovascular accident'/exp
#7. #1 OR #2 OR #3 OR #4 OR #5 OR #6
#6. aids:ab,ti
#5. 'acquired immunodeficiency syndrome':ab,ti
#4. 'acquired immune deficiency syndrome'/exp
#3. 'human immunodeficiency virus':ab,ti
#2. hiv:ab,ti
#1. 'human immunodeficiency virus'/exp

1.1.4: Web of Science: Searched from 1900 to July 29 2015

#1 TOPIC: ("'human immunodeficiency virus") *OR* TOPIC: (hiv)
#2 TOPIC: ("acquired immune deficiency syndrome") *OR* TOPIC: (aids)
#3 #2 OR #1
#4 TOPIC: ("cerebrovascular accident") *OR* TOPIC: (stroke) *OR* TOPIC: (lacunar) *OR* TOPIC: ("intracranial haemorrhage*") *OR* TOPIC: ("atrial fibrillation") *OR* TOPIC: ("vascular disease*") *OR* TOPIC: ("heart infarction" OR "myocardial infarction" OR "heart attack") *OR* TOPIC: (revascularization) *OR* TOPIC: (stent*) *OR*

TOPIC: ("carotid artery disease*") *OR* TOPIC: ("carotid atherosclerosis") *OR* TOPIC: ("heart failure") *OR* TOPIC: ("peripheral occlusive artery disease") *OR* TOPIC: (aorta OR aortic) *OR* TOPIC: (atherosclerosis)
#5 #4 AND #3
#6 TOPIC: (cohort*) *OR* TOPIC: (longitudinal) *OR* TOPIC: (prospective) *OR* TOPIC: ("follow up")
#7 #6 AND #5

1.1.5: LILACS:

(tw:("human immunodeficiency virus" OR hiv OR "acquired immune deficiency syndrome" OR aids)) AND (tw:("cerebrovascular accident" OR stroke OR lacunar OR "intracranial haemorrhage*" OR "atrial fibrillation" OR "vascular disease*" OR "heart infarction" OR "myocardial infarction" OR "heart attack" OR revascularization OR stent* OR "carotid artery disease*" OR "carotid atherosclerosis" OR "heart failure" OR "peripheral occlusive artery disease" OR aorta OR aortic OR atherosclerosis))

1.1.6: Clinicaltrials.gov: Searched 7/29/2015

Studies With Results | Observational Studies | "HIV Infections"
Studies With Results | Observational Studies | "Acquired Immunodeficiency Syndrome"

1.1.7: WHO International Clinical Trials Registry Platform: Searched 7/29/2015

hiv OR "human immunodeficiency virus" OR aids OR "acquired immune deficiency syndrome" in Condition
AND cohort OR longitudinal OR prospective in Title

1.2: Data search detailed methods & Data Collection Process:

Our first search was conducted on PubMed on May 26th, 2015, and 7,203 articles were found. Our second search was carried out in EMBASE, Web of Science and LILACS, within a week (from July 27th to July 31st). 1,172 articles were found to be duplicates from our first search, so we carefully excluded them leaving 2,859 new articles, of which 1,500 were from EMBASE, 1,293 from Web of Science and 66 from LILACS. Our third search was conducted in The Cochrane Library, ClinicalTrials.gov and WHO International Clinical Trials Registry Platform on July 29, 2015, and 248 studies were found. After the exclusion of the non-relevant studies and the duplicates , 37 new articles remained, of which 31 were from The Cochrane Library, 2 from ClinicalTrials.gov and 4 from WHO International Clinical Trials Registry Platform. Therefore, a total of 11,482 studies were screened for inclusion in our meta-analysis (Figure 1).

We obtained from each of the selected articles the demographic, clinical and laboratory data reported by HIV status. We noted whether the study provided a measure of risk (i.e. Hazard ratios, risk ratio, etc.), and whether the given measure was adjusted for confounders. If the study did not report measures of risks, we calculated crude rate ratios with the provided number of events and sample size. We extracted crude incidence rates when provided. If not provided, we estimated incidence rate and their confidence intervals with the data provided by the authors. If the author did not provide total person-year follow-up, we estimated it by multiplying the follow-up time by the number of subjects and contrasted the estimated incidence rate with the reported in the paper. In the

cohorts where HIV negative controls were included, we obtained preferably the adjusted hazard ratios, but if not available, we obtained the unadjusted hazard ratio, the risk ratio, or calculated the unadjusted hazard ratio as indicated elsewhere.[1] We used exchangeable incidence rate ratio (IRR), risk ratio, relative risk, with hazard ratios.[2] We noted whether the population study reflected first-time event, recurrent events in populations with known vascular disease, and the geographical region. Finally, we extracted the hazard ratio or risk ratio in cohorts that included HIV only models.

1.4: Data characterization:

Risk of Bias in Individual Studies: An ideal study would include a random representative sample of the population of HIV in the geographical area of study, compared to a representative or random sample from HIV uninfected controls from the same geographical area. The study must present adjusted hazard ratios by traditional vascular risk factors and ideally would control not only by the presence or absence or vascular risk factors, but also by the degree of control as defined by established clinical guidelines or by using continuous longitudinal measurements of the intensity of such vascular risk factors. A good study would include commodity samples (e.g. administrative datasets, hospital-based, etc.) that included matched (at least by age and sex) HIV- controls, and that reported hazard ratios adjusting for at least demographics and vascular risk factors (expressed categorically or with some continuous measurements measured at baseline). A fair study would report only unadjusted rates of a given outcome.

2.-SUPPORTING RESULTS

Table A: Demographic, clinical and immunological characteristics of HIV cohorts studied at baseline enrollment.															
Ref	Country/ Sample size / sample source	Age	Men (%)	Non-white ethnicity	Hypertension (%)	Diabetes (%)	Dyslipidemia (%)	Smoking (%)	CD4 count	Viral load	ARV use (%)	Illicit drug use (%)	HCV (%)	HBV (%)	Incidence Rates per 1000 person-year (95% CI)
United States															
3-5	US/ 27350/ Administrative datasets (VA)	Mean (SD): 48.2 (9.5) Median: 48	97.3	62.2	Controlled: 7.4 Uncontrolled: 25.4	14	58	60.2	Mean (SD): 390.7 (287.1) Median: 361.5	Mean (SD): 50 317.4 (126 797.8) Median: 680	49.2	11.3	35	*	Death:36.90 (35.90-38.0) AMI 1.94 (1.69-2.20) CHD: 4.67 (4.56-4.79) IS: 2.79 (2.51-3.10)
6	US (CA)/ 28513/ Administrative Dataset (California Medicaid)	18-44y: 69.5% ≥45y: 30.5%	72.7	*	*	*	*	*	*	*	60	*	*	*	**Estimated:** 71,286 py Death: 50.6 (49-52.3) MI: 4.1 (3.7-4.6) CHD: 19.1 (18.1-20.1)
7-10	US (MA) / 4308/ Administrative Dataset (RPDR)	Mean (SD): 41.6 (11.4)	69	47	37	22	40	48	Mean (SD): 473 (317) cells/mm3	Median (IQR): 0 (0, 6.8) log, copies/ml ≤400 copies/ml: 73%	50	*	*	*	AMI: 11.13 (9.58-12.68) IS: 5.27 (4.40-6.20) ICH: 2.29 (1.80-3.00)
11, 12	US (CA)/ 22081/ Administrative Dataset (KPSC, KPNC Health Plans)	Mean (SD): 41 (10)	90.6	44.1	7.3	2.9	5	43.3	Mean: 400 cells/μl	Mean: 55,272 copies/mL	48.7	7.1	*	*	MI: 2.83 (2.50-3.20) IS: 1.25 (1.06-1.46)
13, 14	US/ 1776/ Hospital and community based	Mean (range): 41.0 (17-69	100	42	8.9	1.5	*	45	Mean (IQR): 585 (401) cells/ml	Mean (IQR): 4820 (7,770) copies/mL	100	41	15	*	Stroke: 3.3 (2.5-4.3)
15	US (NC)/ 2216 / Medicaid database	<50: 55.14%	52	82	*	6	11	*	*	*	100	*	*	*	MI: 5.9 (4.3–8.2)
16, 17	US (CA)/ 423 / Hospital-based	Mean 42.3 ± 9.4	81	59	24	9	12	27	Median 274 (range 89-458) cells/mm3	Median 3.7 (range 1.9-4.8) log10 copies/ml	61	30	*	*	**Estimated:** ~ 10582 py All death: 21.9 (19-24.7)
18-21	US (MA)/ 327/ Hospital & community-based	Mean 44±7	74	48	*	9	15	49	Mean 458±299 cells/mm3	Mean 3.1±1.1 copies/mm3	73	5	*	*	**Estimated:** ~ 1014 py All death: 37.5 (26.5-51.0)
22	US (NC)/ 2574/ HIV clinic	Median 39 (IQR 32-45)	98	56	*	*	*	*	Median 466 (IQR 260–700)	> 400 copies/ml: 73%	89	14	16	5	Ischemic stroke: 2.26 (1.53, 3.21)
23	US / 41213 / Hospital Based (VA)	83 % between 35-55 years	98	54	*	*	*	*	32% < 200 CD4 cells/mm3	*	100	32	*	*	Death: 52 (38.80-68.20) in 2003
24	US (NY)/ 311 / Hospital-based	Mean 53± 9	75	*	61	27	39	54	Median 243 (IQR 86-348)	Median 1,240 (IQR 50-	87	*	*	*	Estimated: ~ 901 py

									cells/mm3	36,109)<br>copies/ml					MI 18.9 (11.0-30.2)<br>CHD 34.4 (23.4-48.8)
25	US (CA)/ 5667/<br>HIV-clinic	77% between<br>25-44 years	79	66	*	*	*	*	50% < 200 CD4<br>cells/mm3	14% ≥ 500<br>copies/ml	78	20	*	*	**Estimated:**<br>CAD: 1.6 (1.00-2.40)<br>IS: 2.3 (1.60-3.10)
26, 27	US/ 18603 /<br>HIV clinic	Median 36<br>(range 18-92)	82.6	*	Athena: 1.2<br>HIV Insight: 2.5	Athena:<br>1.06<br>HIV<br>Insight:<br>1.09	*	40.29	46.25% <<br>200cells/µl	*	100	*	*	*	MI: 1.2 (0.98-1.47)<br>Death: 26.38 (25.26-<br>27.55)
28	US (TN)/1129/<br>HIV clinic	Median 40<br>(IQR 34-46)	74	44	*	*	*	*	Median (IQR):<br>243 (110 – 408)	*	100	*	*	*	Death: 34.28<br>(26.6-43.38)
29	US/ 8039/<br>Administrative<br>database (VA)	Mean<br>50.0±7.3	98.2	70.5	HCV+: 32<br>HCV-: 29<br>**WA: 31**	HCV+: 14<br>HCV-: 11<br>**WA: 13**	*	HCV+:20<br>HCV-: 17<br>**WA: 19**	Mean<br>HCV+: 355 ±<br>290 cells/ µl<br>HCV-: 366 ±<br>296 cells/ µl<br>**WA: 358.8**	Mean<br>HCV+: 69812 ±<br>123540<br>copies/µl<br>HCV-: 70143 ±<br>128887 copies/<br>µl<br>**WA: 69927**	*	HCV+:3<br>9<br>HCV-:<br>18<br>**WA:**<br>**31.7**	65.3	*	**Estimated:**<br>~ 39130 py<br>#2503 death<br>Death:<br>64.0 (61.30-66.98)
							**EUROPE**								
30-33	Denmark/<br>5,137/<br>Population-<br>based - Danish<br>HIV Cohort<br>Study	Median (IQR):<br>36 (31.44)	75.8	17.6	4.0	3.0	*	47	Median: 276 (IQR<br>100, 480) cells/ µl	Median 48566<br>copies/ml	77	10.8	16	HBsA<br>g+:<br>6.2	CHD: 4.6 (3.2-6.6)<br>MI: 5.20 (4.25-6.36<br>**Estimated:**<br>Death: 22.8 (21.3-24.3)<br>Any stroke: 3.5 (2.9-4.1)<br>IS 0.94 (0.66-1.29)<br>ICH 0.32 (0.17-0.55)
34	France/ 608/<br>Administrative<br>Dataset (PMSI)	Mean:<br>50.0 ± 10	88.6	*	17.4	9.1	31.1	29.6	*	*	*	*	*	*	**Estimated**: ~ 435 py<br>MI 66.7 (44.68-95.74)<br>CHD 98.9 (71.5-133.2)<br>Any stroke 9.2 (2.5-23.5)
35	France/ 103/<br>Hospital Based<br>(23 French<br>cardiac ICU)	Mean: 48 ±<br>9.1	93	*	18	9	45	59	Median: 462 (270–<br>640) cells.mm3	<200 copies<br>per mL: 69%	96	23	21	9	**Estimated:** ~ 103 py<br>CHD 97.1 (46.6-178.54)
36	Swiss/ 133/<br>Outpatient<br>Clinics	Median (IQR):<br>51 (46-57)	85	*	24.1	13.5	86.5	58.3	Median (IQR): 462<br>(347–701) cell<br>count per mm3	<40 copies per<br>mL: 77%	90	15	*	*	**Estimated:** ~ 133 py<br>Death 37.6 (12.21-87.73)<br>MI 30.1 (8.2-77.0)
37	Denmark/ 647/<br>Hospital based	Median (IQR):<br>37.3 (31.8–<br>45.5)	66.8	29.5	*	*	*	*	Median (IQR): 198<br>(89 – 320) cell<br>count (X10.6<br>cells/L)	Median (IQR):<br>4.8 (4.0-5.3)<br>log10<br>copies/mL	100	5.6	*	*	Death:<br>26.9 (20.2-35.19)
38	Italy/ 201/<br>Outpatient<br>clinic	Mean 45.1 ±<br>10.1	76.1	*	26.4	17.9	*	56.2	Mean 499 ± 283	Undetectable:<br>67%	80	10.5	26.9	*	**Estimated:** 430.5 py<br>Death 27.9 (14.4-48.7)<br>MI 13.9 (5.12-30.34)
39	Swiss/8444/<br>Hospital-based<br>& HIV clinics	Median 45<br>(IQR 39-51)	70.8	*	56.3	4.1	12.7	44.2	Median 350 (IQR<br>180-542) cells/ µl	[On ART]<br><50copies/ml:<br>69.1%<br><50copies/ml:<br>16%	85	IDU:<br>2.6<br>Non-<br>IDU:<br>18	22.7	4	Death: 7.81 (6.74–9.04)<br>MI : 2.44 (1.88–3.18)<br>Cerebral Infarction: 1.73<br>(1.26-2.37)

40	UK/ 7,828/ HIV Clinics	Mean<br>CHD+: 46.6 ± 12.3<br>CHD-: 36.4 ± 8.9<br>**WA:** 36.5	100	CHD+: 20<br>CHD-: 33<br>**WA: 33**	*	*	*	*	Median [cells/mm3]<br>CHD+: 254 (IQR 146–351)<br>CHD-: 283(IQR 169–429) **WA: 283**	*	CHD+: 100<br>CHD-: 72<br>**WA:72**	CHD +: 0<br>CHD-: 4<br>**WA: 4**	CHD +: 21<br>CHD-: 9<br>**WA: 9**	7	CHD: 1.2 (0.8-1.8)
41	France/ 1,173/ Hospital-based	Median<br>MI+: 47 (IQR 41-54)<br>MI-: 46 (IQR 40-54)<br>**WA: 46.25**	89	*	MI+: 20<br>MI-: 12<br>**WA: 13.9**	MI+: 16<br>MI-: 10<br>**WA: 11.5**	MI+: 52<br>MI-: 33<br>**WA: 37.7**	MI+: 64<br>MI-: 40<br>**WA: 45.9**	Median<br>MI+: 427 (IQR 256-638) cells/mm3<br>MI-: 451 (IQR 291-634) cells/mm3<br>**WA: 445**	Median<br>MI+: 127 (IQR 50-3900) copies/ml<br>MI-: 50 (IQR 50-1368) copies/ml<br>**WA: 68.9**	MI+: 95<br>MI-: 93<br>**WA: 93.5**	MI+: 13<br>MI-: 9<br>**WA: 9.9**	*	*	MI: 1.24 (1.11–1.36)
42	Spain/ 125/<br>HIV Clinic	Mean<br>Event: 46±11.01<br>No event: 45±7.94<br>**WA: 45.2**	72<br>77<br>**WA: 76**	*	Event: 20<br>No event: 10<br>**WA: 12**	Event: 8<br>No event: 7<br>**WA: 7.2**	Event: 24<br>No event: 17<br>**WA: 18.4**	88<br>63<br>**WA: 68**	Mean<br>Event: 355±252 cells/mm3<br>No event: 473±242 cells/mm3<br>**WA: 449.4**	[<50copies/ml]<br>Event: 48<br>No event: 82<br>**WA: 75.2**	100	Event: 16<br>No event: 8<br>**WA: 9.6**	*	*	Cerebrovascular Ischemic event: 189 (115-263)
43	Italy/ 1551/ Multicenter Observational Trial	Median 35 (range 22-50)	PI+: 64.9<br>PI-: 63.2<br>**WA: 64**	*	*	*	*	PI+: 46<br>PI-: 48<br>**WA: 47**	Median<br>PI+: 325 (range 170-850) cells/mm3<br>PI-: 350 (range 150-942) cells/mm3<br>**WA: 337.45**	Median<br>PI+: 5.4 (range 3.3-7.5) log10 copies/ml<br>PI-: 5.1 (range 3.2-6.7) log10 copies/ml<br>**WA: 5.25**	100	PI+: 37.9<br>PI-: 36.3<br>**WA: 37.1**	*	*	**Estimated:**<br>~ 4653 py<br>CAD: 5.37 (3.5-7.9)<br>MI 2.8 (1.5-4.8)
44	France/840/ Hospital-based	Mean 43 ± 9.4	*	*	*	*	*	64	Mean 436±233	<2.6log_{10}/ml: 35.2%	85.5	*	*	*	CHD: 5.9 (2.9-10.9)
45	Netherlands/ 3068/ Hospital-based	Median 41 (IQR 35-48)	83.4	*	*	*	*	71.6	Median 360 (IQR 255-510) cells/ µl	Median 5 (IQR 4.6-5.4) log10 copies/ml	100	2.1	4.7	6.3	**Estimated:**<br>10956 py<br>MI 2.0 (1.3-3.0)<br>CHD 3.9 (2.8-5.3)<br>Any stroke 1.3 (0.9-2.1)<br>Any CVD 5.2 (3.9-6.7)
46	France/ 1154/ French centers	37.7 ± 9.5	78	*	*	*	*	15.8	<200 cells/mm3: 35.9%	Detectable: 43.4%	100	17.8	22.4	*	**Estimated:**~ 6500 py<br>MI 4.6 (3.1-6.6)<br>CHD 5.38 (3.75-7.49)<br>Stroke 1.1 (0.4-2.2)<br>ICH 0.3 (0.04-1.11)
47	Spain/ 5185/ HIV care units	Median 36 (IQR 29-43)	79.4	*	*	*	*	*	Median 342 (IQR 163-546) cells/ µl	<10.5copies/ml : 67.6%<br>>10.5copies/ml : 29.4%	0	13.46	11.8	3.65	**Estimated:** 12669 py<br>Death: 13.00 (11.20–15.09<br>MI 1.18 (0.66-1.95)<br>Stroke 0.55 (0.22-1.14)
48, 49	Italy/ 9662/ Database-National AIDS registry	<45: 72%	77.7	*	*	*	*	*	*	*	*	41.8		*	**Estimated:**<br>Death 89.1 (86-92.3)

**LOW-MIDDLE INCOME COUNTRIES**															
50	India/457/<br>Hospital &<br>community-<br>based	≥ 35yo: 43.8<br>%	86	*	*	*	*	*	Median 218<br>(IQR 107–373)<br>cells/ µl	< 10 000<br>copies/ml: 20.6<br>%	30.8<br>5	*	*	*	Death: 13.13 (95% CI;<br>10.46-16.28) per 100 py
51	Ghana/ 4039/<br>HIV Clinic	Median 38<br>(range 14-77)	31.9	*	*	*	*	*	Median 134<br>(range 0-1134)<br>cells/ml	*	100	*	15.2	*	[Crude] Death: 28.83<br>(95% CI; 25.78-32.15)<br>per 1000 py
28	Botswana/<br>650/<br>HIV clinic	Median 33<br>(IQR 29-39)	30.6	100	*	*	*	*	Median 199<br>(IQR 136-252)	*	100	*	*	*	[Crude] CV NADE: 5.0<br>(95% CI; 2.6–9.6) per<br>1,000 py
52	South Africa/<br>15060/ HIV<br>Clinics	≤30y: 18%<br>≥46y: 24%	64	*	*	*	*	*	Median 127<br>(IQR 58-199)<br>cells/ml	Median 4.7<br>(IQR 4.6-5) log<br>10 copies/ml	100	*	*	*	**Estimated:**<br>27873 py<br>Death: 95.4, 91.8-99.1
53	Brazil, Mexico,<br>Peru,<br>Argentina/<br>520/ Dataset -<br>National AIDS<br>programs	*	70	*	*	Event: 11.5<br>No event:<br>3.3<br>**WA: 5.35**	Event: 29.2<br>No event: 21.8<br>**WA: 23.65**	Event:<br>27.7<br>No<br>event:<br>24.4<br>**WA: 25.2**	Event<br>>500cell/µl:<br>17.7%<br><200cell/µl:<br>36.2%<br>No event:<br>>500cell/µl:<br>35.9%<br><200cell/µ:<br>17.2%<br>**WA(>500):**<br>**31.3%**<br>**WA (<200):**<br>**21.9%**	Mean<br>Event: 2.89 ±<br>1.40 log<br>No event: 2.80<br>± 1.29 log<br>**WA: 2.82**	67.7<br><br>69.2<br><br>**WA:**<br>**68.8**	Event:<br>16. 9<br>No<br>event:<br>13.1<br>**WA:**<br>**14.05**	30<br><br>8.5<br><br>**WA:**<br>**13.9**	27.7<br><br>18.2<br><br>**WA:**<br>**20.5**	**Estimated:** ~18300 py<br>MI: 0.6 (0.3-1.08)<br>Stroke: 0.87 (0.50-1.42)
54	Asia-Pacific low<br>income<br>countries/<br>1557/<br>Observational<br>Database	36 (IQR 31-<br>42)	65	*	*	*	*	*	Median 223<br>(IQR 106-358)<br>cells/µl	Median 400<br>(IQR 400–<br>10900)<br>copies/ml	98	5	5	4	**Estimated:**<br>~2691 py<br>Death: 20.8 (15.7-<br>27.02)
**TRANSATLANTIC OR MIXED HIGH INCOME COUNTRIES**															
55-57	Europe, US,<br>Australia/ 33301<br>/ Clinical trial	Median 38<br>(range 3 - 92)	74	46	9	3	38	34	Median<br>(range): 408 (0,<br>2670) cells/µl	≤ 50 copies/ml:<br>33.6 %	67	*	15	5	MI: 3.2 (3.0 -3.4)<br>CHD: 4.8 (4.5-5.0)<br>Stroke: 1.4 (1.2-1.5)<br>CVD: 5.8 (5.5-6.1)<br>Non-hemorrhagic<br>stroke: 0.73 (0.62-0.84)
58	Canada/ 3416/<br>HIV Clinic	Median 36<br>(IQR 31–43)	85	31	11	5	13	49	Median 250<br>(IQR 126–380)<br>cells/mm3	Median 4.7<br>(IQR 4.0–5.2)<br>log10<br>copies/ml	100	5	16	9	Any CVD: 7.59 (95% CI;<br>6.22, 8.96) per 1,000 py<br>Death: 27.3 (95% CI;<br>24.6-29.9) per 1,000 py
59, 60	Canada/ 7,053/<br>Administrative<br>Dataset<br>(RAMQ and<br>Med-Echo)	Mean ± SD:<br>39.5 10.7<br>Median (IQR):<br>37 (22-72)	78	*	23.9	6.6	38.08	*	*	*	76.2	20.2	7.1	*	AMI: 3.88 (3.26-4.58)<br>ICH: 0.83 (0.58-1.19)

61	Australia/ 204/ Hospital-based	Mean CHD+: 50.0 (range 26-70) CHD-: 50.0 (range 26-72) **WA: 50**	94.1	*	CHD+: 41.1 CHD-: 9.5 **WA: 20**	CHD+: 16.1 CHD-: 9.5 **WA: 11.7**	CHD+: 31.3 CHD-: 8.8 **WA: 16.3**	CHD+: 51.4 CHD-: 47.6 **WA: 48.9**	Median CHD+: 481 (range 335–699) cells/ µl CHD-: 426 (range 267–613) cells/ µl **WA: 444.3**	Detectable CHD+: 39% CHD-: 43% **WA: 41.7**	CHD +: 89.8 CHD-: 85.3 **WA: 86.8**	CHD+: 11.7 CHD-: 19.1 **WA: 16.7**	*	*	CHD: 8.5 (6.7-10.9)
54	Australia/Asia-Pacific high income/ 2854/ Observational cohort	Median AHOD: 41 (IQR 35-49) TAHOD HI: 38 (IQR 33-46) **WA: 39**	94 81 **WA: 89**	*	*	*	*	*	Median AHOD: 450 (IQR 270-660) cells/µl TAHOD HI: 290 (IQR 175-437) cells/µl **WA: 386**	Median AHOD: 400 (IQR 400–4467) copies/ml TAHOD HI: 400 (IQR 400-1100) copies/ml **WA: 400**	91 97 **WA: 93**	AHOD:2 TAHOD HI: 3 **WA: 2.4**	7 4 **WA: 5.8**	3 8 **WA: 5**	**Estimated:** ~12385 py Death 12.8 (10.9-15.0)
62	Europe, Israel, Argentina/ 12069/ Hospital-based	Median 38.2 (IQR 32.8-45.3)	74.6	11.4	10.2	2.3	*	41	Median 288 (IQR 162–453) cells/µl	Median 2.84 (IQR 1.69–4.43) log10 copies/ml	100	22	22	5.5	All Death: 18.3 (17.4-19.4)
63	North America, Europe/ 32703/ ART-CC Database	Median HCV+: 39 (IQR 34-44) HCV-: 37 (IQR 31-45) **WA: 37.3**	71 67 **WA: 67.5**	*	*	*	*	*	Median HCV+: 206 (IQR 101-314) cells/ mm3 HCV-: 208 (IQR 93-312) cells/ mm3 **WA: 207.7**	Median HCV+: 4.76 (IQR 3.89-5.27) log copies/mL HCV-: 4.85 (IQR 4.12-5.36) log copies/ mL **WA: 4.83**	100	HCV+:6 2 HCV-: 2 **WA: 10.5**	14.2	*	Death: 1.19 (1.13-1.27)
64	Europe, US/ 201/ PHD - Database	Median 53 (47-62)	89	*	48	18	53	23	Mean 651±510 cell/mm3	<50copies/mm3: 47% <200copies/mm3:9.3%	100	Injection drug: 13 Non-injection: 3.4	24	11	~ 412 py Death: 72.8 (49.1-103.9) MI 48.5 (29.6-74.9) Any stroke 4.9 (0.6-17.5)
65	Europe, Canada, Australia/9858/ CASCADE Database	Median 30 (IQR 25-36)	77	*	*	*	*	*	Median On cART: 324 (IQR 211–475) cells/µl	Median On cART: 4.6 (IQR 3.7-5.2) log10 copies/ml	66.3	15	21	*	Death: 8.38 (7.7-9.1)

Abbreviations: Ref, Reference; HIV, Human Immunodeficiency Virus; US, United States; VA, Veterans Affairs; CI, Confidence Interval; IQR, Interquartile Range; PY, Person-Years; WA, Weighted Average; HBV, Hepatitis B Virus; HBsAg, Surface Antigen of the HBV; ARV, Antiretroviral Drugs; cART, Combination Antiretroviral Therapy; MI, Myocardial Infarction; IS, Ischemic Stroke; CVD, Cardiovascular Disease; CVE, Cerebrovascular Events; CAD, Coronary Artery Disease; PAD, Peripheral Artery Disease; CHF, Congestive Heart Failure; AIDS, Acquired Immunodeficiency Syndrome; CV NADE, Cardiovascular non-AIDS-defining events; HCV+: Hepatitis C infected individuals; TIA, Transient Ischemic Attack; CHD, Coronary Heart Disease; IDU, Injection Drug Use; PI+, Patients receiving 2 nucleoside analogue RTIs (NRTIs) in combination with PIs; PI-, Patients receiving 1 non-nucleoside RTI (NNRTI) in combination with PIs; RPDR, Research Patient Data Registry; KPSC, Kaiser Permanente Southern California; KPNC, Kaiser Permanente Northern California (KPNC); RAMQ, Régie de l'assurance Maladie du Québec; PMSI, Program de Medicalisation des Systèmes d'informatique; AHOD, Australian HIV Observational Database; TAHOD HI, TREAT Asia Observational Database (High income settings); TAHOD LI, TREAT Asia Observational Database (Low income settings); PHD, Percutaneous coronary intervention and surgical revascularization in HIV Database, CASCADE, Concerted Action on SeroConversion to AIDS and Death in Europe; ART-CC, Antiretroviral Therapy Cohort Collaboration; Athena, AIDS Therapy Evaluation Project Netherlands; NC, North Carolina; CA, California; MA, Massachusetts; NY, New York; TN, Tennessee; UK, United Kingdom.

Analytic note: when the data was provided by subgroups within each study, a simple average was used for the purpose of summary quantification. The data was homogenized in unit when possible (i.e. HIV viral load given in 10log was converted to absolute numbers). The indicede rate was converted to fit a 1000 py rate. Person-year follow up were copied from each paper, or calculated based on median follow-up or by approximating it to a reported rate given for other outcomes. Confidence interval were calculated fusing Poisson regression provided by MedCal® Version 16.2.1 64 bit.

Table B: General characteristics of the studies including HIV+ cases compared with HIV- controls.

Study ID	Year(s) of recruitment	City(s)/ Country	Sample size and source of HIV+ cases	Sample size and source of HIV-cases	Follow-up time	Outcomes and ascertainment methods	Measures of risk	Variables used in adjusted analysis	Quality of Study
3	Apr 1, 2003, through Dec 31, 2009	United States	US Veterans: 27,350/ Administrative datasets	US Veterans: 55,109 / Administrative datasets	Median: 5.9 years	**AMI** (Defined using VA, Medicare, and death certificate data)	**Hazard Ratio:** 1.48 (1.27-1.72)	Age, Sex, Race, HTN, DM, LDL, HDL, Smoking, Statin use, EGFR, Anemia, HCV infection, BMI, Cocaine and Alcohol use;	Good
4	Jan 2000 to July 2007		US Veterans: 2,425	US Veterans: 6,154	Median: 7.3 years	**Death (derived from rates)** - (ICD-9 codes)	**Unadjusted Risk Ratio:** 2.08 (1.83-2.36)	*	Fair
66	Oct 1, 2003, to Dec 31, 2009		US Veterans: 25,434	US Veterans: 51,401	Median (IQR): 5.9 (3.5–6.6) years	**Ischemic Stroke**	**Hazard Ratio:** 1.17 (1.01–1.36)	Age, Race, HTN, DM, Dyslipidemia, Smoking, Hepatitis C, GFR, Body mass, Cocaine abuse, Alcohol abuse, Hemoglobin, Atrial Fibrillation.	Good
6	From July 1994 to June, 2000	California/US	28,513 Administrative Data - California Medicaid	3,054,696/ Administrative Data - California Medicaid	[HIV+] Mean (SD): 2.5years (1.8)/ Median: 2.25 years [HIV-] Mean (SD): 2.64years (1.9)/ Median: 2.34 years	**Coronary Heart Disease - Derived** (ICD9-CM)	**Unadjusted Risk Ratio:** 1.02 (0.96-1.07)	*	Fair
						**Death (derived from crude rates)**	**Unadjusted Risk Ratio:** 2.36(2.25-2.48)		
14	July 1, 1996 to June 30, 2011	Baltimore, Chicago, Los Angeles, Pittsburgh/US	1,776/ Hospital and community based	2,169/ Hospital and community based	15year period	**Stroke** (Self-report, prospective active reporting, review of causes of death, or reviewing medical records)	**Risk Ratio**: 2.16 (1.39–3.31)	Adjusted by age (in 10 year intervals), systolic blood pressure, antihypertensive therapy, diabetes mellitus, smoking, and self-reported myocardial infarction.	Fair
11	Jan 1, 1996 to Dec 31, 2009	California/ US	22,081/ Administrative Dataset	230,069/ Administrative Dataset	[HIV+] Mean: 4.5years [HIV-] Mean: 5.4 years	**MI** (ICD-9: 410.x)	**Rate Ratios:** 1.44 (1.27-1.64)	Age, sex, race/ethnicity, calendar era, SES, smoking, overweight, alcohol/drug abuse, diabetes, hypertension and lipid-lowering therapy.	Good
67	Jan 1, 1996 to Dec 31, 2009		24,768/ Administrative Dataset	25,7600 / Administrative Dataset	Up to 835 weeks	**Death (crude rates extracted from clinicaltrials.gov, study ID NCT01339403)**	**Unadjusted Relative Risk:** 5.6 (5.3-5.9)	*	Fair
9	Jan 1, 1996 to Dec 31, 2009	Massachusetts/ US	4,251/ RPDR	35,268/ Research Patient Data Registry (RPDR)	Median: 5.47 years (IQR 1.48, 10.2)	**Intracerebral Hemorrhage -** (ICD-9-CM code)	**Hazard Ratio:** 1.87 (1.23–2.84)	Age, race, hypertension, diabetes, CKD, endocarditis, CNS infections or malignancy, smoking, antiplatelet therapy, anticoagulation, and statin use	Good
68	Jan 1, 1996 to Dec 31, 2009		4,308/ RPDR	32,423/ RPDR	[HIV+] Mean: 5.9 (4.1) years / [HIV] Mean: 6.4 (4.7) years	**Ischemic Stroke** (ICD-9-CM code)	**Hazard Ratio:** 1.21 (1.01-1.46)	Age, gender, race (white vs. other race), hypertension, diabetes, dyslipidemia, smoking (ever vs. never), structural heart disease (at least one diagnosis of cardiomyopathy, left-sided, valvular heart disease, or heart failure), atrial fibrillation/flutter, aspirin use, warfarin use.	Good
7	October 1, 1996 to June 30, 2004		3,851/ RPDR	1,044,589/ RPDR	[HIV+]Mean: 4.5 years / [HIV-]Mean: 3.7 years	**AMI** (ICD-9-CM code)	**Relative Risk:** 1.75 (1.51-2.02)	Age, gender, race, hypertension, diabetes, and dyslipidemia. Not adjusting for smoking	Good
59	Jan 1, 1985 to Dec 31,2007	Canada	7,053/ Administrative Dataset (RAMQ and Med-Echo database)	27,681/ Administrative Dataset (RAMQ and Med-Echo database)	[HIV+] Mean (IQR): 4.2 (1.7–7.4) years [HIV-] Mean (IQR: 2.3 (0.9–5.4) years	**MI** (Identified by the Canadian Classification of Health Interventions CIM-9-QC codes)	**Rate Ratio:** 2.11 (1.69–2.63)	Use of CV-related drugs; prior use of antihypertensive, antidiabetic, lipid-lowering, and antiplatelets, or anti-coagulation drugs; prior history of CVD (myocardial infarction or stroke), chronic renal failure or hemodialysis, hepatitis C infection, illicit drug use, and alcohol abuse.	Good
60	Jan 1, 1985 to Dec 31,2007		7.053	27.681	[HIV+] Mean: 36,144 patient-years [HIV-] Mean: 102,560 patient-years	**Hemorrhagic Stroke** (Defined in the Med-Echo database using ICD- 9 and ICD-10 codes)	**Hazard Ratio:** 3.28 (1.75-6.12)	Adjusted for age, hepatitis C infection, illicit drug use, alcohol abuse, intracranial malignancy or infection, and hemophilia.	Good

	30	From 1 January 1995	Denmark	5,031/ Population-based - Danish HIV Cohort Study	45,279/ Danish Civil Registration System (DCRS)	[HIV+/non-IDU] Median (IQR): 7.6 (3.1–13.4) [HIV+/IDU] Median (IQR): 7.6 (3.1–12.6) [HIV-/non-IDU] Median (IQR): 10.8 (5.7–15.6) [HIV-/IDU] Median (IQR): 15.6 (9.1–15.6)	**Any stroke** (Defined using Danish National Hospital Registry-DNHR)	**Relative Risk:** 1.76 (1.45-2.13))	Country of birth (Denmark vs. outside Denmark) and stratified according to the initial match criteria (age and sex).	Good
							**Hemorrhagic stroke**	**Relative Risk:** 2.12 (1.01-4.42)		
							**Ischemic Stroke**	**Relative Risk:** 1.65 (1.13-2.40)		
	31	From Jan 1, 1995 to Apr 1, 2013		3,233/ Danish HIV Cohort Study	12,932 - Random sample from Copenhagen (CGPS)	[HIV+] 18,263 person-years / [HIV] 63,128 person-years	**MI** (DNHR, coded according ICD-8 and ICD-10)	**Relative Risk:** 2.13 (1.47–3.09)	Gender, age and calendar year, Danish origin, smoking status, body mass index, total cholesterol, diabetes mellitus, and hypertension at baseline	Good
	33	From Jan 1, 1995 to Dec 31, 2004		3,953/ Danish HIV Cohort Study	373,856/ Danish Civil Registration System (DCRS)	HAART period: [HIV+]: 5.23 (2.56–7.55)/ [HIV-]: 5.95 (3.25–7.79)	**Coronary Heart Disease** (DNHR and ICD)	**Relative Risk:** 2.12 (1.63–2.76)	Comorbidities known to be risk factors for ischemic heart disease (diabetes, alcoholism, hypertension, liver disease, and kidney disease)	Good
	32	Jan 1 1995 to Dec 31 2008		5,137/ Population-based - Danish HIV Cohort Study	71,918/ Danish Civil Registration System (DCRS)	[HIV+] Median (IQR): 7.1 years (2.6–12.6) [HIV-] Median (IQR): 10.5years (5.2–14.0)	**Death** (MMR Estimated from the Danish National Registry of Causes of Death)	**Mortality Rate Ratios:** 3.4 (2.5–4.5)	Cohort matched by age and sex, 1:14 ratio.	Fair
	37	Jan 1, 1995 to Jan 2002	Denmark	647/ Hospital based - HIV centers in Western Denmark	64, 700/ Danish Civil Registration System (DCRS)	[HIV+] Median: 3.5 years [HIV-] 215,580 person-years	**Death** (Collected at follow-up visits, Derived from MRR)	**Unadjusted Risk Ratio:** 6.51(3.25-13.02)	*	Fair
	34	Jan 1, 2005, to Dec 31, 2009	France	435/ Administrative Dataset (PMSI) for hospital admissions	945/ Administrative Dataset (PMSI)	For 1 year after discharge	**Recurrent MI** (coded according ICD-10)	**Risk Ratio**: 1.10 (0.71-1.70)	Extracted and Estimated from data assuming time to event= 1 year. Outcomes after MI, hospital based, rates extracted from matched cohorts. Sex and age-adjusted rates)	Fair
							**Any stroke**	**Risk Ratio**: 1.73 (0.46-6.48)		
							**Coronary Heart Disease**	**Risk Ratio**: 1.25 (0.88-1.77)		
	35	September 2003 to March 2006	France	103/ Hospital Based (23 French cardiac ICU)	195/ Hospital Based (23 French cardiac ICU)	Median: 1 year	**Coronary Heart Disease** (events were adjudicated by an independent events committee of cardiologists and then were recorded)	**Unadjusted Hazard Ratio:** 1.4 (0.6-3.0)	*	Fair
	36	Jan 2005 to Dec 2011	Swiss	133/ Outpatient - SHCS	5,328 / Hospital based- AMIS registry	[HIV+] 95%: At least 12 months [HIV-] Median (IQR): 392 (375–414) days	**All Death** (events were collected in specific case reports or questionnaires and then validated)	**Hazard Ratio =:** 4.42 (1.73-11.27)	Gender, age, calendar year of AMI, smoking status, hypertension and diabetes	Good
							**AMI**	**Hazard Ratio:** 1.16 (0.41-3.27)		

Abbreviations: ID, Identification; HIV, Human Immunodeficiency Virus; US, United States; VA, Veterans Affairs; CVD, Cardiovascular Disease; AMI, Acute Myocardial Infarction; IQR, Interquartile Range; SD, Standard Deviation; HTN, Hypertension; DM, Diabetes Mellitus; EGFR, Estimated Glomerular Filtration Rate; HDL, High-Density Lipoprotein; LDL, Low-Density Lipoprotein; HCV, Hepatitis C Virus; BMI, Body Mass Index; IDU, Injection Drug Use; CKD, Chronic Kidney Disease; CNS, Central Nervous System; CV, Cardiovascular; ICU, Intensive Care Unit; HAART, Highly Active Antiretroviral Therapy; ICD-CM, International Classification of Diseases-Clinical Modification; CGPS, Copenhagen General Population Study; PMSI, *Program de Medicalisation des Systèmes d'informatique*; RAMQ, *Régie de l'assurance Maladie du Québec*; SES, Neighborhood Socioeconomic Status; DNHR, Danish National Hospital Registry; SHCS, Swiss HIV Cohort Study; AMIS, Acute Myocardial Infarction in Switzerland Registry; MRR, Mortality Rate Ratio.

Table C: Weighted averages of study characteristics of the HIV samples studied across geographical regions

	United States	Europe	Mixed low to middle income countries	Mixed high-income countries	All regions combined
	(N=sample size/ number of studies)				
**Combined sample size**	164,616/ 15 studies	45,859/ 16 studies	22,283/ 6 studies	101,659/ 9 studies	334,417/ 44 studies*
Mean age (in years)	47.5 (N=42,620/ 7 studies)	38.3 (N=10,859/ 7 studies)	*	39.6 (N=10,111/ 3 studies)	44.66 (N=53,890/ 17 studies)
Median age (in years)	32.1 (N=22,306/ 3 studies)	40.1 (N=19,421/ 8 studies)	37.0 (N=6,246/ 3 studies)	*	36.42 (N=53,890/ 11 studies)
Men (%)	88.7 (N=164,616/ 15 studies)	82.0 (N=45019/ 15 studies)	57.9 (N=22,283/ 6 studies)	73.6 (N=101,659/ 9 studies)	81.1 (333,577/ 43 studies)
Non-white ethnicity (%)	54.3 (N=135,792/ 13 studies)	27.0 (13,612/ 3 study)	*	36.3 (N=48,786/ 3 studies)	48.0 (N=198,190/ 19 studies)
Hypertension (%)	19.9 (N=82,977/ 8 studies)	33.6 (9,943/ 4 studies)	*	11.4 (56,244/ 6 studies)	18.9 (N=155,145/ 18 studies)
Diabetes (%)	8.1 (N=85,520/ 10 studies)	4.8 (N=15,924/ 8 studies)	5.0 (N=520/ 1 studies)	3.4 (N=56,244/ 6 studies)	3.8 (N=102,673/ 18 studies)
Dyslipidemia (%)	33.6 (N=57,016/ 7 studies)	18.1 (10,586/ 6 studies)	24.0 (N=520/ 1 studies)	36.0 (N=44,175/ 5 studies)	33.0 (N=112,297/ 19 studies)
Smoking (%)	47.4 (N=83,304/ 9 studies)	48.1 (N=22,537/ 12 studies)	25.0 (520/ 1 studies)	36.8 (N=49,191/ 5 studies)	41.0 (N=175,758/ 27 studies)
CD4 mean (cells/mm3)	401.6 (N=63,967/ 6 studies)	448 (N=1,166/ 3 studies)	*	651 (N=201/ 1 study)	403 (N=65,334/ 10 studies)
CD4 median (cells/mm3)	363 (N=31,787/ 5 studies)	323 (N=33,269/ 6 studies)	139.2 (N=21,763/ 5 studies)	308.3 (N=94,405/ 7 studies)	300 (N=181,224/ 22 studies)
Viral load mean (copies/μl)	53,029 (N=59,659/5 studies)	NA	NA	NA	53,029 (N=59,659/5 studies)
Viral load median (copies/μl)	652 (N=32,392/ 4 studies)	75,414 (N=11,576/ 5 studies)	44,100 (N=17,137/ 3 studies)	454,716 (N=60,900/ 5 studies)	36,342 (N=122,005/ 17 studies)
cART use (%)	70.3 (N=136.845/ 9 studies)	67.0 (N=23,211/ 8 studies	90.6 (N=21,633/ 5 studies)	80.7 (N=101,659/ 9 studies)	75.13 (N=289,368/ 31 studies)
Illicit drug use (%)	20.0 (N=101,497/ 6 studies)	16.3 (N=33,118/ 10 studies)	7.3 (N=2,077/ 2 studies)	13.8 (N=68,358/ 8 studies)	17.23 (N=205,050/ 26 studies)
Hepatitis C coinfection (%)	39.0 (N=39,825/ 4 studies)	14.7 (N=31,120/ 8 studies)	12.4 (N=6,116/ 3 studies)	15.3 (N=101,455/ 8 studies)	20.4 (N=178,516/ 23 studies)
Hepatitis B coinfection (%)	5.0 (N=5,990/ 1 study)	5.5 (N=9,765/ 6 studies)	8.3 (N=2,077/ 2 studies)	4.6 (N=61,699/ 5 studies)	5.0 (N=93,541/ 14 studies

Abbreviations: cART, combined antiretroviral therapy; NA, not data available.
*Two studies included cohorts in different regions and were included in their respective geographic region.

Table D: Difference in demographic and clinical characteristics in HIV+ compared with HIV- cases.*

Ref	Age (year)	Men	Non-white ethnicity	Dyslipidemia	HTN	DM	Smoking	drug use	Baseline cardiac disease	Hepatitis C	Statin Use	Antiplatelet/ Anticoagulant use	Afib/ Flutter
	Population-based cohort/ community--based												
(Rasmussen et al., 2011; Rasmussen et al., 2015)	-0.2	+22.2	+20.0	*	+0.5	+1.7	+27.5	0.0	*	*	*	*	*
(Mateen et al., 2013; Mateen et al., 2012)	-2.5	0.0	+16.0		+4.0	-0.01	+7.0	+8.0	+1.3	+8.0	*	*	*
(Jensen-Fangel et al., 2004)	*	*	*	*	*	*	*	*	*	*	*	*	*
	Administrative datasets												
(Freiberg et al., 2013; Freiberg et al., 2011)	-0.6	+0.1	0.0	+12.0	-8.8	-6.7	+6.2	+4.1	*	+19.4	-3.3	*	-0.17
(Currier et al., 2003)	<44y: + 11%	+41.0	*	*	*	*	*	*	*	*	*	*	*
(Chow et al., 2012; Triant, Lee, Hadigan, & Grinspoon, 2007)	-1.0	+28.7	+12.0	+5.7	+5.3	+4.9	+18	*	+4	*	- 2.0	+2.0	*
(Silverberg et al., 2014)	<44 y: -1.0	+0.1	-10.1	*	-0.8	+0.2	+14.3	+11.9	*	*	*	*	*
(Durand, Sheehy, Baril, Lelorier, & Tremblay, 2011, 2013)	-0.2	0.0	*	+6.8	+8.8	+1.9	*	+17.9	0.0	+6.8	*	+5.67	*
	Recurrent events- Hospital based												
(Lorgis et al., 2013)	-18.3	+22.3	*	+2.1	-4.7	-1.6	-0.6	*	+3.4	*	*	*	-1.9
(Carballo et al., 2015; Schoeni-Affolter et al., 2010)	-13.0	+12.8	*	-7.9	-36.6	-2.1	+18.7	*		*	*	-6.8	*
(Boccara et al., 2011)	-2.0	-1.0	*	-1	-6	-3	-5	+17.0	*	*	-2.0	0.0	*
	Summary												
Weighted average ± SD	-1.0 ± 0.3	35.2 ± 9.0	7.6 ± 3.0	5.7 ± 3.0	3.9 ± 1.3	3.8 ± 1.2	17.3 ± 4.4	11.7 ± 3.0	3.9 ± 1.2	9.5 ± 1.8	-2.0 ± 0.6	2.1 ± 0.6	-0.05 ± 0.1

Analytic note: We used the earliest reference reported for a given sample, or the sample that reported the largest number or participants. If a given variable was not reported in one paper, then it was extracted from the next chronological paper that reported it. The result from each study was weighted by the sample size to obtain the final summary measures.

Table E: All-death risks among HIV+ samples

Study ID	Measure of association	Age (years)	Man	Ethnicity	Dyslipidemia	HTN	DM	Smoking	CD4 count at death	ARV	Viral load	Drug Use	HBV Coinfection	HCV Coinfection
20	**Unadjusted [*] Hazard Ratios**	By 10-year increase: 1.44 (0.93-2.23) *	1.30 (0.61-2.78)*	White: 1.12 (0.59-2.12)*	Total Cholesterol, by 10 mg/dl increase: 0.91 (0.84-0.98)*<br>HDL: 0.81 (0.64-1.02)*<br>LDL: 0.88 (0.80-0.97)*	Systolic BP by 10mmHg increase: 1.08 (0.90-1.30)* Diastolic BP by 10mmHg increase: 0.92 (0.68-1.25)*	*	2.73 (1.35-5.52)*	<200 cell/ul 3.96 (2.03-7.71)*	*	>100k 7.26 (3.64-14.48)*	5.38 (2.24-12.94)*	*	*
23	**Adjusted Hazard Ratios**	*	*	*	*	*	*	*	*	NNRTI or a PI: 0.18 (0.15 to 0.23) PI: 0.21 (0.15 to 0.28)	*	*	*	
39	**Adjusted Hazard Ratios**	50–64 y: 1.67 (1.19–2.33) / ≥ 65y: 6.25 (3.90–10.0)	*	*	*	*	*	2.40 (1.53–3.76)	Square-root CD4 count: 0.880 (0.858–0.903)	*	*	2.78 (1.93–4.02)	*	
29	**Adjusted Hazard Ratios**	Per 10-year: 1.32 (1.21-1.44)	0.98 (0.55-1.73)	Black: 0.86 (0.74-1)<br>Hispanic: 0.79 (0.61-1.03)	*	0.98 (0.85-1.14)	1.45 (1.22-1.71)	0.97 (0.82-1.14)	0.9 (0.88-0.93) per 100 cells/µL	*	*	1.06 (0.9-1.26)	*	1.58 (1.36-1.84)
50	**Adjusted Relative Risk**	≥35y: 1.97 (1.23-3.14)	1.83 (0.57-5.92)	*	*	*	*	*	201- 350cells/µL: 4.82 (1.03-22.49)<br>101-200cells/µL: 7.72 (1.69-35.19)<br>≤100 cells/µL: 33.20 (7.59, 145.29)<br>**Derived <200: 16.34 (5.67-47.08)**	ART naive: 5.60 (3.18-9.86)	≥10 000 copies/ml: 1.34 (0.52-3.47)	*	*	*
63	**Adjusted Hazard Ratios**	*	*	*	*	*	*	*	*	*	*	1.57 (1.27-1.94)	*	2.04 (1.68-2.47)
4	**Adjusted Hazard Ratios**	*	*	*	*	*	*		*	PI: 0.97 (0.88-1.07) NNRTI : 0.97 (0.88-1.07) NRTI: 0.69 (0.63-0.76)	*	*	*	*
51	**Unadjusted [*] and Adjusted Hazard Ratios**	≥ 40y: 0.94 (0.75–1.17)*	1.69 (1.29–2.21)	*	*	*	*	*	< 200 cell/ul: 2.39 (1.64–3.49)	D4T plus 3TC: 1.60 (1.21–2.11) Efavirenz: 1.15 (0.87–1.51)	*	*	*	*
69	**Adjusted Relative Risk**	Per year: 1.06 (1.03-1.09)	1.30 (0.91-1.86)	*	*	*	*	*	*	*	*	*	*	*
52	**Unadjusted [*] and Adjusted Hazard Ratios**	Per 5-year: 1.1 (1.1–1.1)	1.2 (1.0–1.3)	*	*	*	*	*	≤50cells/mm3: 5.2 (4.2–6.4)<br>51-100 cells/mm3: 2.8 (2.2–3.4)<br>101-200 cells/mm3: 1.7 (1.4–2.1)<br>201-350 cells/mm3: 1.2 (0.94-1.5)<br>**Derived: <200: 2.97, 2.64-3.34**	*	>4.7 HIV RNA log10: 1.6 (1.4–1.7)*	*	*	*
37	**Adjusted Relative Risk**	≥ 40 years: 3.59 (1.81 – 7.12)	1.07 (0.48 – 2.37)	Caucasian: 1.59 (0.55 – 4.59)	*	*	*	*	100-199 x 10.6 cells/L: 1.43 (0.52 – 3.94)<br>50-99 x 10.6 cells/L: 3.44 (1.41 – 8.42)<br><50 x 10.6 cells/L: 3.55 (1.46 – 8.65)<br>**Derived: <200: 2.72, 1.59-4.65**	*	≥100 000 copies/mL: 1.95 (0.96 – 3.95)	1.95 (0.76 – 5.01)	*	*

Abbreviations: ID, Identification; HIV, Human Immunodeficiency Virus; HTN, Hypertension; DM, Diabetes Mellitus; BP, Blood Pressure; HBV, Hepatitis B Virus; HCV, Hepatitis C Virus; HDL, High-Density Lipoprotein; LDL, Low-Density Lipoprotein; ARV, Antiretroviral Drugs; IV, Intravenous; PI, Protease Inhibitor; NNRTI, Non-nucleoside Reverse Transcriptase Inhibitors; NRTIs, Nucleoside Reverse Transcriptase Inhibitors; D4T, Stavudine; 3TC, Lamivudine; RNA, Ribonucleic Acid; Y, Years.

Table F: Myocardial infarction risks among HIV+ individuals

Study ID	Measure of association	Age (years)	Man	Ethnicity	Dyslipidemia	HTN	DM	Smoking	CD4 count	ARV	Viral load	IV Drug Use	HBV Coinfection	HCV Coinfection
**55, 70, 71**	**Adjusted Hazard Ratios and Adjusted Relative Rate [#]**	Per 5 years: 1.34 (1.27–1.40)	1.93 (1.36–2.74)	*	Total cholesterol (per mmol/l higher): 1.28 (1.22–1.34)	Systolic BP by 10mmHg increase: 1.04 (1.00–1.08)	2.28 (1.73–3.01)	Past: 2.01 (1.41–2.86)<br>Current: 4.02 (2.96-5.46)	CD4 nadir < 100: 1.36 (0.57-3.23)<br>100-199: 1.17 (0.50-2.74)<br>Ref: 200-299<br>Latest CD4: <100: 0.96 (0.62-1.50)<br>100-200: 0.95 (0.68-1.33)<br>Ref: 200-299<br>**WA: <200:0.95, 0.73-1.25**	cART: 1.16 (1.02-1.33)<br>Abacavir, current exposure: 2.04 (1.66–2.51)<br>(aRR) Per year of exposure to PI: 1.16 (1.10-1.23) #	At baseline (per log10) 0.94 (0.75-1.18) #<br>Peak: 1.11 (0.92-1.34)	*	Inactive : 1.07 (0.79–1.43)#<br>Active : 0.78 (0.52–1.15)#	0.86 (0.62–1.19)#
**15**	**Adjusted Hazard Ratio**	*	*	*	*	*	*	*	*	Abacavir: 2.05 (0.72–5.86, compared to receiving tenofovir as backbone)	*	*	*	*
**72**	**Adjusted Hazard Ratios**	Per 10 years, 1.79, (1.60-2.01)	*	*	*	2.05 (1.57-2.67)	1.01 (0.76-1.35)	1.01 (0.78-1.30)	*	Per year of use: 1.12 (1.01-1.25)	*	*	*	1.25 (0.98–1.61)
**41, 73, 74**	**Adjusted Relative Hazard**	Per 10 years increase: 1.42 (1.10–1.85)	*	*	*	*	*	*	*	[NRTIs]: 0.93 (0.19–4.65)<br>[NNRTIs]: 1.38 (0.67–2.83)	*	*	*	*
**26, 75**	**Adjusted Hazard Ratios**	35-50years: 2.59 (1.51–4.43)<br>50–65 years: 3.62 (1.77–7.43)<br>≥ 65 years: 8.88 (1.94–40.61)	2.64 (1.06–6.55)	*	1.70 (0.63–4·60)	2.01 (0·71–5·66)	5·11 (1.79–14·6)	Ever: 2.25 (1.20–4.21)	*	PI (per year): 1.19 (1.01–1.40)<br>NNRTI (years): 0.93 (0.71–1.21)<br>PI + NNRTI (years): 0.94 (0.63–1.39)<br>PI (any): 6.51 (0.89-47.8)<br>Any cART: 1.26 (1.07-1.48)	*	*	*	*
**76**	**Adjusted Hazard Ratio**		*	*	*	2.06 (1.40–3.01)	*	*	*	*	*	*	*	*
**11**	**Adjusted Rate Ratios**	40-49y: 3.2 (1.80-5.71)<br>50-64y: 5.94 (3.35-10.56)<br>≥ 65y: 11.87 (6.29-22.42)	1.89 (1.01-3.53)	Black: 0.60 (0.41-0.87)<br>Hispanic: 0.62 (0.41-0.93)<br>Other: 0.95 (0.51-1.75)	Prior lipid-lowering therapy: 1.61 (1.21-2.13)	1.99 (1.51-2.61)	1.53 (1.10-2.12)	2.21 (1.66-2.93)	Per 100 cells/µL: 1.03 (0.97-1.10)	Any cART: 1.26 (0.83-1.91)	HIV RNA (per 1 log): 1.03 (0.97-1.08)	0.68 (0.36-1.31)	*	*
**64**	**Adjusted Hazard Ratios**	*	*	*	*	*	*	*	<200 cells/mm3: 5.9 (1.4-25.0)	Not being treated with NRTIs: 2.6 (0.5-13.5)	*	*	*	*
**7**	**Adjusted Relative Risk**	*	*	African American: 1.43 (1.01-2.00)	3.65 (2.59-5.19)	1.23 (0.90-1.68)	1.33 (0.95-1.85)	*	*	*	*	*	*	*
**31**	**Adjusted Incidence Rate Ratio**	*	*	*	*	*	*	Previous: 2.64 (1.16-6.01)<br>Current: 6.06 (2.99-12.25)	*	*	*	*	*	*
**39**	**Adjusted Hazard Ratio**	Ref < 50 years<br>50–64 y: 5.95 (3.12-11.3) /<br>≥ 65y: 5.89 (2.17-16.0)	*	*	*	*	*	*	*	*	*	*	*	*

Abbreviations: ID, Identification; HIV, Human Immunodeficiency Virus; HTN, Hypertension; DM, Diabetes Mellitus; HBV, Hepatitis B Virus; HCV, Hepatitis C Virus; ARV, Antiretroviral Drugs; IV, Intravenous; BP, Blood Pressure; PI, Protease Inhibitor; NNRTI, Non-nucleoside Reverse Transcriptase Inhibitors; NRTIs, Nucleoside Reverse Transcriptase Inhibitors; cART, Combination Antiretroviral Therapy; RNA, Ribonucleic Acid; Ref, Reference; WA, Weighted Average; Y, Years.

Table G: Coronary Heart Disease risks among HIV+ individuals

Study ID	Measure of association	Age (years)	Male sex	Ethnicity	Dyslipidemia	HTN	DM	Smoking	CD4 count	ARV	Viral load	IV Drug Use	HBV Coinfection	HCV Coinfection
55, 70	**Adjusted Hazard Ratios and Adjusted Relative Rate [#]**	Per 5 years: 1.41 (1.35–1.46)	1.76 (1.33–2.32)	*	Total cholesterol (per mmol/l higher): 1.24 (1.19–1.30)	Systolic BP by 10mmHg increase: 1.04 (1.00–1.07)	1.93 (1.52–2.44)	Past: 1.62 (1.23–2.12)	CD4 nadir < 100: 0.98 (0.45-2.16)[#]<br>100-199: 0.89 (0.41-1.93)[#]<br>Ref: 200-299<br>Latest CD4:<br><100: 0.89 (0.30-2.63)[#]<br>100-199: 0.99 (0.76-1.29)[#]<br>Ref: 200-299	Abacavir: 1.73 (1.45–2.06)	*	*	*	*
77	**Adjusted Hazard Ratios**	*	*	*	*	*	*	*	*	Abacavir: 1.43 (0.96–2.13)<br>Tenofovir: 0.87 (0.53–1.42)	*	*	*	*
4	**Adjusted Hazard Ratios**	*	*	*	*	*	*	*	*	PI use: 2.15 (0.99-4.65)<br>NNRTI use: 1.40 (0.70-2.77)	HIV-1 RNA >500 1.29 (0.68-2.45	*	*	*
40	**Unadjusted [*] and Adjusted Incidence Rate Ratios**	Per 10 years: 2.81 (1.51–5.25)	*	Black: 0.61 (0.17–2.17)	*	*	*	*	Per 50 cell increase: 1.02 (0.93–1.12)*	On cART: 0.81 (0.33–1.99)*<br>Abacavir use: 0.94 (0.30–2.99)*<br>Tenofovir use: 1.18 (0.33–4.14)	HIV RNA > 400 copies/mL: 0.37, (0.13-1.12)	*	HBsAg+: 0.77 (0.99–5.88)*	HCV Ab +: 3.94 (1.00–15.5)
43	**Unadjusted [*] Relative risk**	*	*	*	*	*	*	*	*	PI use: 11.5 (2.72-48.55)*	*	*	*	*
6	**Adjusted Relative Risk**	*	*	*	18-33y: 3.91 (2.67-5.72)<br>34-49 y: 3.01 (2.56-3.54)<br>50-65y: 2.58 (2.04-3.27)<br>≥ 66: 1.76 (1.06-2.95)<br>**WA: 2.88 (2.55-3.25)**	18-33y: 5.33 (3.68-7.73)<br>34-49 y: 5.00 (4.26-5.87)<br>50-65y: 3.51 (2.73-4.53)<br>≥ 66: 4.50 (2.50-8.10)<br>**WA:4.60 (4.06-5.21)**	18-33y: 1.78 (1.25-2.52)<br>34-49 y: 1.66 (1.42-1.94)<br>50-65y: 1.67 (1.32-2.12)<br>≥ 66: 1.31 (0.80-2.17)<br>**WA:1.68 (1.49-1.89)**	*	*	18-33y: 2.06 (1.42-2.99)<br>34-49 y: 1.08 (0.91-1.28)<br>50-65y: 0.79 (0.63-1.00)<br>≥ 66: 1.15 (0.65-2.04)<br>**WA: 1.06 (0.94-1.21)**	*	*	*	*

Abbreviations: ID, Identification; HIV, Human Immunodeficiency Virus; HTN, Hypertension; DM, Diabetes Mellitus; HBV, Hepatitis B Virus; HCV, Hepatitis C Virus; ARV, Antiretroviral Drugs; cART, Combination Antiretroviral Therapy; IV, Intravenous; PI, Protease Inhibitor; NNRTI, Non-nucleoside Reverse Transcriptase Inhibitors; RNA, Ribonucleic Acid; HBsAg, Surface Antigen of the HBV; HCV Ab, Anti-HCV Antibody; WA, Weighted Average; Ref, Reference; Y, Year.

Table H: Cerebrovascular risks among HIV+ individuals

Study ID	Outcome/ Measure of association	Age (years)	Male sex	Ethnicity	Dyslipidemia	HTN	DM	Smoking	CD4 count	ARV	Viral load	IV Drug Use	HBV Coinfection	HCV Coinfection
55	**Any stroke/ Adjusted Relative Rate**	Per 5 years older: 1.41 (1.35, 1.49)	1.19 (0.88, 1.62)	*	*	2.14 (1.66, 2.75)	*	1.52 (1.12, 2.06)	CD4 nadir < 100: 1.36 (0.54-3.43)<br>100-199: 1.54, 0.63-3.79<br>Ref: 200-299<br>Latest CD4: <100: 2.26, 1.29-3.94<br>100-200: 1.63, 1.03-2.59<br>Ref: 200-299		*	*	*	*
22	**Any stroke/ Adjusted Incidence Rate Ratio**	Per 10 year: 1.78 (1.25, 2.55)	0.59 (0.23-1.53)	Black: 0.89 (0.46, 1.74)	3.02 (1.48-6.17)	1.96 (0.99-3.99)	0.95 (0.42, 2.18)	0.81 (0.40, 1.65)	< 200 cells/mm3: 2.83 (1.27-6.33)	4.16 (0.80-21.65)	> 400 copies/mL: 3.97 (1.90-8.31)	*	*	1.81 (0.85, 3.84)
72, 77	**Any stroke/ Adjusted Hazard Ratios**	1.65 (1.54-1.76)	*	*	*	1.48 (1.28-1.75)	*	*	*	Abacavir: 2.10 (1.20-3.66)<br>Tenofovir 0.67 (0.31-1.42)	*	*	*	1.20 (1.04–1.38)
30	**Any stroke/ Adjusted Incidence Rate Ratio**	*	*	*	*	*	*	*	≤200cells/µL and non-HAART: 2.26 (1.05 – 4.86)<br>≤200cells/µL and HAART: 1.17 (0.50 – 2.75)<br>>200cells/µL and HAART: 0.80 (0.47 – 1.36)	*	*	*	*	*
14	**Any stroke/ Adjusted Relative Risk**	Per 10-year: 1.80 (1.26-2.55)	*	*	*	SBP: 1.16, (1.10-1.24)<br>LVH: 2.20, (1.26-3.84)	1.41 (0.97-2.04)	1.69 (1.27-2.23)	*	*	*	*	*	*
39	**Ischemic stroke/ Adjusted Hazard Ratios**	50–64 y: 3.96 (1.86–8.42) / ≥ 65y: 17.7 (7.06–44.5)	*	*	*	*	*	*	*	*	*	*	*	*
10	**Ischemic stroke/ Adjusted Hazard Ratios**	Per year: 1.06 (1.03-1.09)	1.03 (0.53-2.00)	0.71 (0.41-1.25)	0.99 (0.55-1.80)	0.79 (0.44-1.45)	0.59 (0.28-1.23)	0.83 (0.49-1.40)	CD4 nadir (per 50): 0.97 (0.90 -1.05)	NRTI use: 1.19 (0.51 - 2.79)<br>NNRTI use: 0.38 (0.19 - 0.76)<br>PI use: 0.63 (0.30 -1.33)	1.10 (1.04-1.17) per log	*	*	*
12	**Ischemic stroke/ Adjusted Hazard Ratios**	>40 year: 4.54 (3.02-6.83)	0.77 (0.41-1.45)	Black 1.4 (1.0-2.1)<br>All Non_white: 1.11 (0.83-1.49)	1.4 (0.9-2.0)	3.3 (2.2-4.7)	1.4 (0.9-2.1)	1.0 (0.7-1.4)	<200 vs >500: 2.5 (1.3-4.6)<br>CD4 nadir < 200: 0.8 (0.4-1.6)	Any cART: 1.2 (0.7-1.9)	10 000 vs. <500: 1.5 (0.9-2.4)	2.0 (1.5-2.9)	*	*
9	**Intracranial hemorrhage/ Adjusted Hazard Ratios**	*	0.56 (0.24-1.28)	*	*	*	*	*	< 200: 4.61 (2.09–10.17)	0.46 (0.18–1.15)	*	*	*	*

Abbreviations: ID, Identification; HIV, Human Immunodeficiency Virus; HTN, Hypertension; DM, Diabetes Mellitus; HBV, Hepatitis B Virus; HCV, Hepatitis C Virus; ARV, Antiretroviral Drugs; IV, Intravenous; HAART, Highly Active Antiretroviral Therapy; SPB, Systolic Blood Pressure; LVH, Left Ventricular Hypertrophy; PI, Protease Inhibitor; NNRTI, Non-nucleoside Reverse Transcriptase Inhibitors; NRTIs, Nucleoside Reverse Transcriptase Inhibitors; Ref, Reference; Y, Years.

Table I: Vascular death risks among HIV+ individuals

Study ID	Outcome/ Measure of association	Age (years)	Man	Ethnicity	Dyslipidemia	HTN	DM	Smoking	CD4 count	ARV	Viral load	IV Drug Use	HBV Coinfection	HCV Coinfection
**48**	**Vascular Death (by MI, CHD, DM) / Crude Risk ratio**	≥ 45 years: 0.74 (0.3-1.48)	1.80 (0.88-3.67)	*	*	*	*	*	*	*	*		*	*
**64**	**Cardiac Death/ Adjusted Hazard Ratios**	*	*	*	*	*	*	*	<200 cells/mm3: 1.4 (0.3-8.3)	Not being treated with NRTIs: 9.9 (2.1-46)	*	*	*	*
**65**	**Cardiovascular Death/ Adjusted Hazard Ratios**	>35 years: 3.61 (1.69 – 7.72)	*	*	*	*	*	*	349-200 cells/μL: 1.15 (0.51 – 2.63)<br>199-50 cells/μL: 0.89 (0.28 – 2.82)<br><50cells/μL: 4.15 (1.14 – 15.17)<br>**Derived <200: 1.76, 0.74-4.17**	*	≥5 HIV RNA log10: 4.69 (2.04-10.79)	*	*	*
**62**	**Cardiovascular Death/ Adjusted Incidence Rate Ratios**	*	*	*	*	*	*	*	*	By year longer on cART: 0.99 (0.93-1.06)<br>By cumulative exposure to cART:<br>< 2 years: 0.45 (0.25–0.83)<br>4-5.99 years: 0.97 (0.57–1.63)<br>6-7.99 years: 1.05 (0.61–1.80)<br>>8 years: 1.11 (0.66–1.86)	*	*	*	*
**16**	**Sudden Cardiac Death/ Adjusted Hazard Ratios**	*	*	*	*	*	*	*	*	*	Ejection Fraction < 40%, VL <50: 2.7 (0.3 - 27.6)<br>Ejection Fraction < 40%, VL >50: 11.7 (2.9 - 47.2)	*	*	*

Abbreviations: ID, Identification; HIV, Human Immunodeficiency Virus; HTN, Hypertension; DM, Diabetes Mellitus; HBV, Hepatitis B Virus; HCV, Hepatitis C Virus; ARV, Antiretroviral Drugs; cART, Combination Antiretroviral Therapy; VL, Viral Load; IV, Intravenous; CHD, Coronary Heart Disease; MI, Myocardial Infarction; NRTIs, Nucleoside Reverse Transcriptase Inhibitors; RNA, Ribonucleic Acid.

Table J: Any vascular disease risks among HIV+ individuals

Study ID	Outcome	Measure of association	Age (years)	Man	Ethnicity	Dyslipidemia	HTN	DM	Smoking	CD4 count	ARV	Viral load	IV Drug Use	HBV Coinfection	HCV Coinfection
70	**Cardiovascular Disease** (MI, Stroke, Invasive CV procedures)	**Adjusted Hazard Ratios**	Per 5 years older: 1.42 (1.37–1.47)	1.70 (1.32–2.18)	*	Total cholesterol (per mmol/l higher): 1.21 (1.16–1.27)	Systolic BP by 10mmHg increase: 1.05 (1.03–1.08)	1.92 (1.55–2.38)	Past: 1.27 (1.00–1.61)	*	Abacavir: 1.63 1.38–1.92	*	*	*	*
58	**Cardiovascular Disease** (CHD, MI, CHF, Cerebrovascular accident or Stroke; Coronary bypass; Angioplasty, Sudden cardiac death)	**Unadjusted [*] and Adjusted Hazard Ratios**	Per 10 years: 1.99 (1.69-2.33)	2.00 (0.92-4.34)	Black: 0.64 (0.28-1.46)<br>Aboriginal: 0.98 (0.54-1.79)<br>Other: 1.20 (0.66-2.18)	*	*	*	Past: 1.34 (0.78- 2.33)<br>Current: 1.50 (1.00-2.26)	Per 100 cells/ mm3: 0.97 (0.88-1.04)*	[Initiation] 1990-1995: 0.32 (0.18-0.58)<br>1995-2000: 0.25 (0.14-0.46)<br>2000-2005: 0.16 (0.07-0.38)<br>>2005: 0.21 (0.08-0.58)	*	1.17 (0.75-1.81)*	1.05 (0.63-1.74)	1.44 (0.97-2.13)
38	**Vascular Event** (AMI, TIA, Stroke, Death)	**Adjusted Hazard Ratios**	Per 5 years increase: 1.55 (1.19–2.02)	*	*	Total cholesterol, 10 mg/dL increase: 1.06 (0.98–1.14)	0.44 (0.13–1.50)	*	3.87 (1.25–12.0)	*	*	*	*	*	*
26	**AtD events** **(**Angina, MI, CHD, Cerebrovascular Accident, TIA, PAD, other CV events)	**Adjusted Hazard Ratios**	35–50years: 3.23 (2.34–4.45)<br>50–65 years: 7.26 (4.99–10.56)<br>≥ 65 years: 18.11 (9.32–35.21)	1.00 (0.72–1.40)	*	*	Pre-ART hypertension: 3.26 (2.20–4.82)<br>Systolic BP ≥ 140 mmHg: 0.99 (0.50–1.96)	Pre-ART DM: 2.54 (1.49–4.33)	Ever: 1.40 (1.06–1.86)	*	PI (years): 1.03 (0.95–1.13)<br>NNRTI (years): 0.91 (0.80–1.04)<br>PI + NNRTI (years): 1.09 (0.88–1.34)	*	*	*	*
78	**Atherosclerotic Cardiovascular Disease** (CHD, Peripheral Vascular Disease, Cerebrovascular Disease)	**Adjusted Hazard Ratios**	Per 5-year increase: 1.10 (1.05 -1.15)	*	African American: 0.61 (0.46-0.82)	*	*	1.42 (1.05-1.91)	*	*	*	*	*	*	*
79	**MI, unstable angina and ischemic stroke**	**Unadjusted [*] and Adjusted Relative Hazard**	*	*	African American: 0.73 (0.37-1.45)<br>Latino: 1.61 (0.84-3.07)<br>Other: 2.88 (1.05- 7.88)	1.86 (1.26- 2.76)	2.07 (1.47-2.91)	1.40 (0.94-2.08)	Current: 1.66 (1.08-2.54)<br>Past: 1.49 (1.03- 2.16)	≤50: 0.63 (0.19-2.04)*<br>51-200: 0.55 (0.24-1.24)*<br>201-350: 1.23 (0.72-2.10)*<br>351-500: 1.19 (0.70, 2.02)*	Years on HAART (per 1 year increase): 1.11 (1.03-1.20)	Per 1 log: 0.86 (0.71-1.04)	*	*	*
45	**Cardiovascular events** (Coronary bypass, coronary stenting and/ or angioplasty, fatal and nonfatal MI and CVA)	**Adjusted Hazard Ratios**	Per 1-year: 1.10 (1.07 – 1.13)	*	*	*	*	*	*	200–350 cells/μL: 0.34 (0.14 – 0.86)<br>351–500 cells/μL: 0.62 (0.29 – 1.31)<br>>500 cells/μL: 0.52 (0.22 – 1.20)	*	*	*	*	*
46	**Major CADE** (MI, Stroke, CHD, PAD and CV surgery for coronary disease)	**Unadjusted [*] and Adjusted Hazard Ratios**	1.07 (1.04–1.10)	4.00 (1.24-12.88)	*	*	*	*	4.19 (2.17–8.11)	<200 cells/mm: 0.99 (0.55–1.80)*	ART adherence: 2.42 (1.17–5.02)*<br>Antiretroviral Naivety: 1.14 (0.64–2.02)*	Detectable viral load: 0.99 (0.53–1.84)*	0.92 (0.50–1.70)*	*	1.16 (0.59–2.29)*
47	**Cardiovascular Events** (AMI, Angina, CHF, Stroke, TIA, Silent Cerebrovascular Disease, PAD, Coronary-related death)	**Adjusted Incidence Rate Ratios**	41-50y: 5.36 (2.07- 13.90)<br>>50y: 5.61 (2.29-13.74)	*	*	*	*	*	*	350-500cells/μL: 0.34 (0.10- 1.12)<br>200-350cells/μL: 0.85 (0.31- 2.27)<br><200 cells/μL: 1.76 (0.54- 5.68)	1.11 (0.27- 4.44)	>10.5 copies/ml: 1.05 (0.51-2.17)	*	*	*
25	**Cardiovascular Disease** (CHD, Stroke)	**Adjusted Hazard Ratios**	2.16 (1.75-2.66)	1.19 (0.62-2.26)	Black: 1.59 (0.9-2.81)<br>Hispanic: 0.88 (0.48-1.61)<br>Other: 1.28 (0.44-3.72)	*	*	*	*	[Lowest Observed] 1.04 (0.50-2.18)<br>[Highest Observed] 0.80 (0.40-1.61)	PI use: 6.22 (3.13-12.39)<br>Non-PI ART use: 3.18 (1.99-5.09)	*	*	*	*

Abbreviations: Abbreviations: ID, Identification; HIV, Human Immunodeficiency Virus; HTN, Hypertension; DM, Diabetes Mellitus; HBV, Hepatitis B Virus; HCV, Hepatitis C Virus; ARV, Antiretroviral Drugs; IV, Intravenous; CV, Cardiovascular; CHD, Coronary Heart Disease; MI, Myocardial Infarction; CHF, Congestive Heart Failure; TIA, Transient Ischemic Attack; PAD, Peripheral Artery Disease; CVA, Cerebrovascular Attack; CABG, Coronary Artery Bypass; CADE, Major Coronary or Other Arterial Disease Event; BP, Blood Pressure; PI, Protease Inhibitor; NNRTI, Non-nucleoside Reverse Transcriptase Inhibitors; ART, Antiretroviral Therapy; HAART, Highly Active Antiretroviral Therapy; Y, Years.

Table K: Other Vascular Outcomes risks among HIV+ individuals

Study ID	Outcome	Measure of association	Age (years)	Man	Ethnicity	Dyslipidemia	HTN	DM	Smoking	CD4 count	ARV		Viral load	IV Drug Use	HBV Coinfection	HCV Coinfection
77	Heart failure	Adjusted Hazard Ratios	*	*	*	*	*	*	*	*	Abacavir: 1.45 (0.85–2.47	Tenofovir: 1.82 (1.02–3.24)	*	*	*	*
	Peripheral arterial disease		*	*	*	*	*	*	*	*	Abacavir: 1.00 (0.41–2.46)	Tenofovir: 0.70 (0.25–1.98)	*	*	*	*
39	Coronary Angioplasty	Adjusted Hazard Ratios	50–64 y: 4.72 (2.76–8.10)<br>≥ 65y: 7.43 (3.51–15.7)	*	*	*	*	*	*	*	*		*	*	*	*
26	AtD or death	Adjusted Hazard Ratios	35-50years: 1.46 (1.33–1.60)	1.10 (0.98–1.25)			Pre-ART hypertension : 1.70 (1.36–2.12)<br>Systolic BP ≥ 140 mmHg: 0.90 (0.65-1.24)	Pre-ART Diabetes: 1.67 (1.24–2.26)	Ever: 0.97 (0.88–1.08)		PI (years): 0.76 (0.73–0.78)		*	*	*	*
			50–65 years: 2.11 (1.84–2.41)								NNRTI (years): 0.69 (0.65–0.74)					
			≥ 65 years: 4.51 (3.22–6.31)								PI + NNRTI (years): 1.77 (1.61–1.95)					
80	Cardiac Death in patient with cardiomyopathy	Unadjusted [*] Hazard Ratios	Per year: 0.99 (0.93–1.06)*	*	*	0.64 (0.17–2.41)*	0.68 (0.20–2.22)*	0.83 (0.18–3.85)*	*	1.00 (0.99–1.00) per cell/ mm3*	*		1.00(1.00-1.00) per copy/ml*	*	*	*
57	Composite end point of sudden death or the first non-hemorrhagic stroke	Adjusted Rate Ratio	Per year of age: 1.07 (1.06-1.08)	*	*	*	1.60 (1.19-2.15)	1.50 (1.05-2.15)	Current: 1.62 (1.13-2.32)	*	Current or Recent PI exposure: 1.22 (0.95-1.57) Cumulative exposure to PI (per year): 1.06 (1.01- 1.11)		*	*	*	*
54	Deaths from non-AIDS causes (CVD related, liver related, non-AIDS cancers, (un) natural, bacterial infection related)	Unadjusted [*] and Adjusted Hazard Ratios	30-39y: 1.18 (0.56 – 2.49)	1.32, (0.76-2.28)	*	*	*	*	*	101-200 cells/μL: 1.61 (0.96 – 2.69)	*		401-10 000 copies/ml: 1.25 (0.67 – 2.31)	1.46 (0.60 – 3.55)	1.38 (0.66 – 2.87) *	1.57 (0.88 – 2.79) *
			40-49y: 1.68 (0.80 – 3.55)							≤100cells/μ : 3.71 (2.36 – 5.83)			≥ 10 001 copies/ml: 1.28 (0.77-2.14)			
			>50y: 4.08 (1.95 – 8.54)													
63	MI/IHD as Cause of Death	Adjusted Hazard Ratios	*	*	*	*	*	*	*	*	*		*	*	*	0.91 (0.12-6.85)
	Stroke as Cause of Death													0.71 (0.06-8.57)		3.15 (0.65-15.38)
	Other heart/vascular disease as Cause of Death													1.38 (0.45-4.23)		2.78 (1.05-7.38)

Abbreviations: ID, Identification; HIV, Human Immunodeficiency Virus; HTN, Hypertension; DM, Diabetes Mellitus; HBV, Hepatitis B Virus; HCV, Hepatitis C Virus; ARV, Antiretroviral Drugs; IV, Intravenous; AtD, Atherosclerotic Disease; ART, Highly Active Antiretroviral Therapy; PI, Protease Inhibitor; NNRTI, Non-nucleoside Reverse Transcriptase Inhibitors; MI, Myocardial Infarction; IHD, Ischemic Heart Disease; AIDS, Acquired Immunodeficiency Syndrome; BP, Blood Pressure; CVD, Cardiovascular Disease; Y, Years.

Note: The unadjusted Hazard Ratios were marked with asterisks.

Table L: Characteristics of the HIV studies from the United States included in this study and the NHANES-derived characteristics of HIV infected individuals in the United states and the general uninfected population.

	Published prospective studies from the United Stated on HIV and vascular disease. Mean ± SD	NHANES data from 20-59 year old 2009-2014*	
		HIV+ population Mean ± SE	HIV – population Mean ± SE
	N= 164,616	N=62	N=10291
Mean age (in years)	47.5 ± 8.1	43.7 ± 2.0	39.5 ± 0.25
Men (%)	88.7 ± 7.7	80.9 ± 4.3	49.5 ± 0.5
Non-white ethnicity (%)	54.3 ± 7.4	64.6 ± 9.3	36.1 ± 2.1
Uninsured (%)	Not available	20.6 ± 5.3	24.4 ± 0.9
Hypertension (%)	19.9 ± 0.8	35.2 ± 8.9	27.1 ± 0.6
Diabetes (%)	8.1 ± 3.1	6.1 ± 4.0	7.2 ± 0.3
Obesity (%)	Not available	22 ± 5.0	35.6 ± 0.7
Dyslipidemia (%)	33.6 ± 7.1	39.4 ± 9.3	32.5 ± 0.7
Smoking (%)	47.4 ± 5.9	50.1 ± 7.9	22.7 ± 0.8
cART use (%)	70.3 ± 8.1	56.2 ± 9.3	na
Illicit drug use (%)	20.0 ± 3.3	44.0 ± 10.4	20.9 ± 0.9
Hepatitis C co-infection (%)	39.0 ± 6.8	14.7 ± 8.4	1.9 ± 0.3
Hepatitis B co-infection (%)	5.0	3.2 ± 2.0 (38.2 ± 8.9 past exposure)	0.4 ± 0.01 (4.0 ± 0.3 past exposure)

*NHANES limited testing HIV serology to age range 20-59 years old, and for cART use, Hepatitis C co-infection and Hepatitis B infection, NHANES data was only available from 2009-2012. NHANES analytic note: Risk factors definition: Hypertension (HTN) was defined by the use of antihypertensive mediations, and/or a mean systolic blood pressure (average of an initial and subsequent reading during the in-person examination) ≥ than 140 mmHg, and/or a mean diastolic blood pressure ≥ than 90 mmHg, and/or a physician's diagnosis of high blood pressure. Diabetes mellitus (DM) was defined as the presence of glycosylated hemoglobin ≥ than 6.5 % and/or physician's diagnosis of DM. Dyslipidemia was recorded as present if participants responded 'Yes' to the question: "Have you ever been told by a doctor or other health professional that your blood cholesterol level was high?". Obesity was defined as a body mass index ≥ 30. Smoking was defined as individuals who reported smoking more than 100 cigarettes in their life time and who currently smoke either some or all days. Cocaine use was defined as present if it occurred over the year prior to the interview. HIV status in NHANES was determined by a positive ELISA HIV test in blood. The use of combined antiretroviral therapy was determined by the use of any combined antiretroviral therapy listed in the medication log capture by the NHANES interviewer. For the prevalence of vascular risks factors, the estimates were weighted to account for oversampling and non-response. We used survey procedures to obtain the means and their standard errors. The analysis was carried out with SAS software, version 9.4 (SAS Institute Inc., Cary, NC).

REFERENCES:

1. Parmar MK, Torri V and Stewart L. Extracting summary statistics to perform meta-analyses of the published literature for survival endpoints. *Stat Med*. 1998;17:2815-34.
2. Hernán MA. The Hazards of Hazard Ratios. *Epidemiology (Cambridge, Mass)*. 2010;21:13-15.
3. Freiberg MS, Chang CC, Kuller LH, Skanderson M, Lowy E, Kraemer KL, Butt AA, Bidwell Goetz M, Leaf D, Oursler KA, Rimland D, Rodriguez Barradas M, Brown S, Gibert C, McGinnis K, Crothers K, Sico J, Crane H, Warner A, Gottlieb S, Gottdiener J, Tracy RP, Budoff M, Watson C, Armah KA, Doebler D, Bryant K and Justice AC. HIV infection and the risk of acute myocardial infarction. *JAMA Intern Med*. 2013;173:614-22.
4. Freiberg MS, Chang CC, Skanderson M, McGinnis K, Kuller LH, Kraemer KL, Rimland D, Goetz MB, Butt AA, Rodriguez Barradas MC, Gibert C, Leaf D, Brown ST, Samet J, Kazis L, Bryant K, Justice AC and Veterans Aging Cohort S. The risk of incident coronary heart disease among veterans with and without HIV and hepatitis C. *Circ Cardiovasc Qual Outcomes*. 2011;4:425-32.
5. Sico JJ, Chang CC, So-Armah K, Justice AC, Hylek E, Skanderson M, McGinnis K, Kuller LH, Kraemer KL, Rimland D, Bidwell Goetz M, Butt AA, Rodriguez-Barradas MC, Gibert C, Leaf D, Brown ST, Samet J, Kazis L, Bryant K and Freiberg MS. HIV status and the risk of ischemic stroke among men. *Neurology*. 2015;84:1933-40.
6. Currier JS, Taylor A, Boyd F, Dezii CM, Kawabata H, Burtcel B, Maa JF and Hodder S. Coronary heart disease in HIV-infected individuals. *J Acquir Immune Defic Syndr*. 2003;33:506-12.
7. Triant VA, Lee H, Hadigan C and Grinspoon SK. Increased acute myocardial infarction rates and cardiovascular risk factors among patients with human immunodeficiency virus disease. *J Clin Endocrinol Metab*. 2007;92:2506-12.
8. Triant VA, Regan S, Lee H, Sax PE, Meigs JB and Grinspoon SK. Association of immunologic and virologic factors with myocardial infarction rates in a US healthcare system. *J Acquir Immune Defic Syndr*. 2010;55:615-9.
9. Chow FC, He W, Bacchetti P, Regan S, Feske SK, Meigs JB, Grinspoon SK and Triant VA. Elevated rates of intracerebral hemorrhage in individuals from a US clinical care HIV cohort. *Neurology*. 2014;83:1705-11.
10. Chow FC, Regan S, Feske S, Meigs JB, Grinspoon SK and Triant VA. Comparison of Ischemic Stroke Incidence in HIV-Infected and Non–HIV-Infected Patients in a US Health Care System. *J Acquir Immune Defic Syndr*. 2012;60:351–358.
11. Silverberg MJ, Leyden WA, Xu L, Horberg MA, Chao CR, Towner WJ, Hurley LB, Quesenberry CP, Jr. and Klein DB. Immunodeficiency and risk of myocardial infarction among HIV-positive individuals with access to care. *J Acquir Immune Defic Syndr*. 2014;65:160-6.
12. Marcus JL, Leyden WA, Chao CR, Chow FC, Horberg MA, Hurley LB, Klein DB, Quesenberry CP, Jr., Towner WJ and Silverberg MJ. HIV infection and incidence of ischemic stroke. *AIDS*. 2014;28:1911-9.
13. Mateen FJ, Shinohara RT, Carone M, Miller EN, McArthur JC, Jacobson LP and Sacktor N. Neurologic disorders incidence in HIV+ vs HIV- men: Multicenter AIDS Cohort Study, 1996-2011. *Neurology*. 2012;79:1873-80.
14. Mateen FJ, Post WS, Sacktor N, Abraham AG, Becker JT, Smith BR, Detels R, Martin E, Phair JP and Shinohara RT. Long-term predictive value of the Framingham Risk Score for Stroke in HIV-positive vs HIV-negative men. *Neurology*. 2013;81:2094-102.
15. Brouwer ES, Napravnik S, Eron JJ, Jr., Stalzer B, Floris-Moore M, Simpson RJ, Jr. and Sturmer T. Effects of combination antiretroviral therapies on the risk of myocardial infarction among HIV patients. *Epidemiology*. 2014;25:406-17.
16. Moyers BS, Secemsky EA, Vittinghoff E, Wong JK, Havlir DV, Hsue PY and Tseng ZH. Effect of left ventricular dysfunction and viral load on risk of sudden cardiac death in patients with human immunodeficiency virus. *Am J Cardiol*. 2014;113:1260-5.
17. Tseng ZH, Secemsky EA, Dowdy D, Vittinghoff E, Moyers B, Wong JK, Havlir DV and Hsue PY. Sudden Cardiac Death in Patients With Human Immunodeficiency Virus Infection. *J Am Coll Cardiol*. 2012;59:1891-1896.

18. Lai H, Fishman EK, Gerstenblith G, Moore R, Brinker JA, Keruly JC, Chen S, Detrick B and Lai S. Vitamin D deficiency is associated with development of subclinical coronary artery disease in HIV-infected African American cocaine users with low Framingham-defined cardiovascular risk. *Vasc Health Risk Manag*. 2013;9:729-37.
19. Forrester J, Spiegelman D, Woods M, Knox T, Fauntleroy J and Gorbach S. Weight and body composition in a cohort of HIV-positive men and women. *Public Health Nutr*. 2001;4:743-747.
20. Mangili A, Polak JF, Quach LA, Gerrior J and Wanke CA. Markers of atherosclerosis and inflammation and mortality in patients with HIV infection. *Atherosclerosis*. 2011;214:468-73.
21. Volpe GE, Tang AM, Polak JF, Mangili A, Skinner SC and Wanke CA. Progression of carotid intima-media thickness and coronary artery calcium over 6 years in an HIV-infected cohort. *J Acquir Immune Defic Syndr*. 2013;64:51-7.
22. Vinikoor MJ, Napravnik S, Floris-Moore M, Wilson S, Huang DY and Eron JJ. Incidence and clinical features of cerebrovascular disease among HIV-infected adults in the Southeastern United States. *AIDS Res Hum Retroviruses*. 2013;29:1068-74.
23. Bozzette SA, Ake CF, Tam HK, Phippard A, Cohen D, Scharfstein DO and Louis TA. Long-term survival and serious cardiovascular events in HIV-infected patients treated with highly active antiretroviral therapy. *J Acquir Immune Defic Syndr*. 2008;47:338-41.
24. Wever Pinzon O, Silva Enciso J, Romero J, Makani H, Fefer J, Gandhi V, Bangalore S and Chaudhry FA. Risk stratification and prognosis of human immunodeficiency virus-infected patients with known or suspected coronary artery disease referred for stress echocardiography. *Circ Cardiovasc Imaging*. 2011;4:363-70.
25. Vaughn G and Detels R. Protease inhibitors and cardiovascular disease: analysis of the Los Angeles County adult spectrum of disease cohort. *AIDS Care*. 2007;19:492-9.
26. Kwong GP, Ghani AC, Rode RA, Bartley LM, Cowling BJ, da Silva B, Donnelly CA, van Sighem AI, Cameron DW, Danner SA, de Wolf F and Anderson RM. Comparison of the risks of atherosclerotic events versus death from other causes associated with antiretroviral use. *AIDS*. 2006;20:1941-50.
27. Zuniga J. *A decade of HAART : the development and global impact of highly active antiretroviral therapy*. Oxford; New York: Oxford University Press; 2008.
28. Wester CW, Koethe JR, Shepherd BE, Stinnette SE, Rebeiro PF, Kipp AM, Hong H, Bussmann H, Gaolathe T, McGowan CC, Sterling TR and Marlink RG. Non-AIDS-defining events among HIV-1-infected adults receiving combination antiretroviral therapy in resource-replete versus resource-limited urban setting. *AIDS*. 2011;25:1471-1479.
29. Erqou S, Mohanty A, Murtaza Kasi P and Butt AA. Predictors of mortality among united states veterans with human immunodeficiency virus and hepatitis C virus coinfection. *ISRN Gastroenterology*. 2014;2014.
30. Rasmussen LD, Engsig FN, Christensen H, Gerstoft J, Kronborg G, Pedersen C and Obel N. Risk of cerebrovascular events in persons with and without HIV: a Danish nationwide population-based cohort study. *AIDS*. 2011;25:1637-46.
31. Rasmussen LD, Helleberg M, May MT, Afzal S, Kronborg G, Larsen CS, Pedersen C, Gerstoft J, Nordestgaard Bo G and Obel N. Myocardial infarction among danish HIV-infected individuals: Population-attributable fractions associated with smoking. *Clin Infect Dis*. 2015;60:1415-1423.
32. Helleberg M, Kronborg G, Larsen CS, Pedersen G, Pedersen C, Gerstoft J and Obel N. Causes of death among Danish HIV patients compared with population controls in the period 1995-2008. *Infection*. 2012;40:627-634.
33. Obel N, Thomsen HF, Kronborg G, Larsen CS, Hildebrandt PR, Sorensen HT and Gerstoft J. Ischemic heart disease in HIV-infected and HIV-uninfected individuals: a population-based cohort study. *Clin Infect Dis*. 2007;44:1625-31.
34. Lorgis L, Cottenet J, Molins G, Benzenine E, Zeller M, Aube H, Touzery C, Hamblin J, Gudjoncik A, Cottin Y and Quantin C. Outcomes after acute myocardial infarction in HIV-infected patients: analysis of data from a French nationwide hospital medical information database. *Circulation*. 2013;127:1767-74.

35. Boccara F, Mary-Krause M, Teiger E, Lang S, Lim P, Wahbi K, Beygui F, Milleron O, Gabriel Steg P, Funck-Brentano C, Slama M, Girard PM, Costagliola D, Cohen A and Prognosis of Acute Coronary Syndrome in HIVipI. Acute coronary syndrome in human immunodeficiency virus-infected patients: characteristics and 1 year prognosis. *Eur Heart J*. 2011;32:41-50.
36. Carballo D, Delhumeau C, Carballo S, Bahler C, Radovanovic D, Hirschel B, Clerc O, Bernasconi E, Fasel D, Schmid P, Cusini A, Fehr J, Erne P, Keller PF, Ledergerber B, Calmy A, Aubert V, Barth J, Battegay M, Bernasconi E, Boni J, Bucher HC, Burton-Jeangros C, Calmy A, Cavassini M, Egger M, Elzi L, Fehr J, Fellay J, Francioli P, Furrer H, Fux CA, Gorgievski M, Gunthard H, Haerry D, Hasse B, Hirsch HH, Hirschel B, Hosli I, Kahlert C, Kaiser LK, Keiser O, Kind C, Klimkait T, Kovari H, Ledergerber B, Martinetti G, Martinez de Tejada B, Metzner K, Muller N, Nadal D, Pantaleo G, Rauch A, Regenass S, Rickenbach M, Rudin C, Schmid P, Schultze D, Schoni-Affolter F, Schupbach J, Speck R, Taffe P, Tarr P, Telenti A, Trkola A, Vernazza P, Weber R and Yerly S. Increased mortality after a first myocardial infarction in human immunodeficiency virus-infected patients; a nested cohort study. *AIDS Res Ther*. 2015;12:4.
37. Jensen-Fangel S, Pedersen L, Pedersen C, Larsen CS, Tauris P, Moller A, Sorensen HT and Obel N. Low mortality in HIV-infected patients starting highly active antiretroviral therapy: a comparison with the general population. *AIDS*. 2004;18:89-97.
38. Parruti G, Vadini F, Sozio F, Mazzott E, Ursini T, Polill E, Di Stefano P, Tontodonati M, Verrocchio MC, Fulcheri M, Calella G, Santilli F and Manzoli L. Psychological factors, including alexithymia, in the prediction of cardiovascular risk in HIV infected patients: results of a cohort study. *PLoS One*. 2013;8:e54555.
39. Hasse B, Ledergerber B, Furrer H, Battegay M, Hirschel B, Cavassini M, Bertisch B, Bernasconi E and Weber R. Morbidity and aging in HIV-infected persons: the Swiss HIV cohort study. *Clin Infect Dis*. 2011;53:1130-9.
40. Campbell LJ, Desai M, Hegazi A, Ibrahim F, Melikian N, Hay P, Fox JM and Post FA. Renal impairment is associated with coronary heart disease in HIV-positive men. *HIV Clin Trials*. 2012;13:343-9.
41. Lang S, Mary-Krause M, Cotte L, Gilquin J, Partisani M, Simon A, Boccara F and Costagliola D. Impact of individual antiretroviral drugs on the risk of myocardial infarction in human immunodeficiency virus-infected patients: a case-control study nested within the French Hospital Database on HIV ANRS cohort CO4. *Arch Intern Med*. 2010;170:1228-38.
42. Corral I, Quereda C, Moreno A, Perez-Elias MJ, Dronda F, Casado JL, Muriel A, Masjuan J, Alonso-de-Lecinana M and Moreno S. Cerebrovascular ischemic events in HIV-1-infected patients receiving highly active antiretroviral therapy: incidence and risk factors. *Cerebrovasc Dis*. 2009;27:559-63.
43. Barbaro G, Di Lorenzo G, Cirelli A, Grisorio B, Lucchini A, Hazra C and Barbarini G. An open-label, prospective, observational study of the incidence of coronary artery disease in patients with HIV infection receiving highly active antiretroviral therapy. *Clin Ther*. 2003;25:2405-18.
44. Escaut L, Monsuez JJ, Chironi G, Merad M, Teicher E, Smadja D, Simon A and Vittecoq D. Coronary artery disease in HIV infected patients. *Intensive Care Med*. 2003;29:969-73.
45. Van Lelyveld SFL, Gras L, Kesselring A, Zhang S, De Wolf F, Wensing AMJ and Hoepelman AIM. Long-term complications in patients with poor immunological recovery despite virological successful HAART in Dutch ATHENA cohort. *AIDS*. 2012;26:465-474.
46. Carrieri MP, Protopopescu C, Le Moing V, Reboud P, Raffi F, Mahy S, Roux P, Cuzin L, Spire B, Leport C and Grp ACA-CS. Impact of immunodepression and moderate alcohol consumption on coronary and other arterial disease events in an 11-year cohort of HIV-infected patients on antiretroviral therapy. *BMJ open*. 2012;2.
47. Masia M, Padilla S, Alvarez D, Lopez JC, Santos I, Soriano V, Hernandez-Quero J, Santos J, Tural C, del Amo J, Gutierrez F and CoRis. Risk, predictors, and mortality associated with non-AIDS events in newly diagnosed HIV-infected patients: role of antiretroviral therapy. *AIDS*. 2013;27:181-189.
48. Serraino D, Bruzzone S, Zucchetto A, Suligoi B, De Paoli A, Pennazza S, Camoni L, Dal Maso L, De Paoli P and Rezza G. Elevated risks of death for diabetes mellitus and cardiovascular diseases in Italian AIDS cases. *AIDS Res Ther*. 2010;7.
49. Serraino D, Zucchetto A, Suligoi B, Bruzzone S, Camoni L, Boros S, De Paoli A, Dal Maso L, Franceschi S and Rezza G. Survival after AIDS diagnosis in Italy, 1999-2006: a population-based study. *J Acquir Immune Defic Syndr*. 2009;52:99-105.

50. Ghate M, Deshpande S, Tripathy S, Godbole S, Nene M, Thakar M, Risbud A, Bollinger R and Mehendale S. Mortality in HIV infected individuals in Pune, India. *Indian J Med Res*. 2011;133:414-420.

51. Sarfo FS, Sarfo MA, Norman B, Phillips R, Bedu-Addo G and Chadwick D. Risk of deaths, AIDS-defining and non-AIDS defining events among Ghanaians on long-term combination antiretroviral therapy. *PLoS One*. 2014;9.

52. Hoffmann CJ, Fielding KL, Johnston V, Charalambous S, Innes C, Moore RD, Chaisson RE, Grant AD and Churchyard GJ. Changing Predictors of Mortality Over Time From cART Start: Implications for Care. *Jaids-Journal of Acquired Immune Deficiency Syndromes*. 2011;58:269-276.

53. Belloso WH, Orellana LC, Grinsztejn B, Madero JS, La Rosa A, Veloso VG, Sanchez J, Moreira RI, Crabtree-Ramirez B, Messina OG, Lasala MB, Peinado J and Losso MH. Analysis of serious non-AIDS events among HIV-infected adults at Latin American sites. *HIV Med*. 2010;11:554-564.

54. Falster K, Choi JY, Donovan B, Duncombe C, Mulhall B, Sowden D, Zhou J and Law MG. AIDS-related and non-AIDS-related mortality in the Asia-Pacific region in the era of combination antiretroviral treatment. *AIDS*. 2009;23:2323-2336.

55. Sabin CA, Ryom L, De Wit S, Mocroft A, Phillips AN, Worm SW, Weber R, D'Arminio Monforte A, Reiss P, Kamara D, El-Sadr W, Pradier C, Dabis F, Law M, Lundgren J and Group DADS. Associations between immune depression and cardiovascular events in HIV infection. *AIDS*. 2013;27:2735-48.

56. Sabin CA, Ryom L, Kovari H, Kirk O, de Wit S, Law M, Reiss P, Dabis F, Pradier C, El-Sadr W, Monforte A, Kamara D, Phillips AN and Lundgren JD. Association between ALT level and the rate of cardio/cerebrovascular events in HIV-positive individuals: the D: A: D study. *J Acquir Immune Defic Syndr*. 2013;63:456-63.

57. Worm SW, Kamara DA, Reiss P, Fontas E, De Wit S, El-Sadr W, D'Arminio Monforte A, Law M, Phillips A, Ryom L, Dabis F, Weber R, Sabin C, Lundgren JD and Group DADS. Evaluation of HIV protease inhibitor use and the risk of sudden death or nonhemorrhagic stroke. *J Infect Dis*. 2012;205:535-9.

58. Gillis J, Smieja M, Cescon A, Rourke SB, Burchell AN, Cooper C, Raboud JM and Group OCS. Risk of cardiovascular disease associated with HCV and HBV coinfection among antiretroviral-treated HIV-infected individuals. *Antivir Ther*. 2014;19:309-17.

59. Durand M, Sheehy O, Baril JG, Lelorier J and Tremblay CL. Association between HIV infection, antiretroviral therapy, and risk of acute myocardial infarction: a cohort and nested case-control study using Quebec's public health insurance database. *J Acquir Immune Defic Syndr*. 2011;57:245-53.

60. Durand M, Sheehy O, Baril JG, LeLorier J and Tremblay CL. Risk of spontaneous intracranial hemorrhage in HIV-infected individuals: a population-based cohort study. *J Stroke Cerebrovasc Dis*. 2013;22:e34-41.

61. Trevillyan JM, Cheng AC and Hoy J. Abacavir exposure and cardiovascular risk factors in HIV-positive patients with coronary heart disease: a retrospective case-control study. *Sex Health*. 2013;10:97-101.

62. Kowalska JD, Reekie J, Mocroft A, Reiss P, Ledergerber B, Gatell J, d'Arminio Monforte A, Phillips A, Lundgren JD, Kirk O and Euro Ssg. Long-term exposure to combination antiretroviral therapy and risk of death from specific causes: no evidence for any previously unidentified increased risk due to antiretroviral therapy. *AIDS*. 2012;26:315-23.

63. May MT, Justice AC, Birnie K, Ingle SM, Smit C, Smith C, Neau D, Guiguet M, Schwarze-Zander C, Moreno S, Guest JL, Monforte AD, Tural C, Gill MJ, Bregenzer A, Kirk O, Saag M, Sterling TR, Crane HM and Sterne JAC. Injection Drug Use and Hepatitis C as Risk Factors for Mortality in HIV-Infected Individuals: The Antiretroviral Therapy Cohort Collaboration. *J Acquir Immune Defic Syndr*. 2015;69:348-354.

64. D'Ascenzo F, Cerratoa E, Appleton D, Moretti C, Calcagno A, Abouzaki N, Vetrovec G, Lhermusier T, Carrie D, Das Neves B, Escaned J, Cassese S, Kastrati A, Chinaglia A, Belli R, Capodanno D, Tamburino C, Santilli F, Parodi G, Vachiat A, Manga P, Vignali L, Mancone M, Sardella G, Fedele F, DiNicolantonio JJ, Omede P, Bonora S, Gaita F, Abbate A, Zoccai GB and Percutaneous Coronary I. Prognostic Indicators for Recurrent Thrombotic Events in HIV-infected Patients with Acute Coronary Syndromes: Use of Registry Data From 12 sites in Europe, South Africa and the United States. *Thromb Res*. 2014;134:558-564.

65. Marin B, Thiebaut R, Bucher HC, Rondeau V, Costagliola D, Dorrucci M, Hamouda O, Prins M, Walker S, Porter K, Sabin C and Chene G. Non-AIDS-defining deaths and immunodeficiency in the era of combination antiretroviral therapy. *AIDS*. 2009;23:1743-1752.

66. Sico JJ, Chang CCH, So-Armah K, Justice AC, Hylek E, Skanderson M, McGinnis K, Kuller LH, Kraemer KL, Rimland D, Bidwell Goetz M, Butt AA, Rodriguez-Barradas MC, Gibert C, Leaf D, Brown ST, Samet J, Kazis L, Bryant K and Freiberg MS. HIV status and the risk of ischemic stroke among men. *Neurology*. 2015;84:1933-1940.

67. Kaiser Permanente HIV Cohort Study. 2016;2016.

68. Chow FC, Regan S, Feske S, Meigs JB, Grinspoon SK and Triant VA. Comparison of ischemic stroke incidence in HIV-infected and non-HIV-infected patients in a US health care system. *Journal of acquired immune deficiency syndromes*. 2012;60:351-8.

69. Zaccarelli M, Gattari P, Rezza G, Conti S, Spizzichino L, Vlahov D, Ippolito G, Lelli V and Valenzi C. Impact of HIV infection on non-AIDS mortality among Italian injecting drug users. *AIDS*. 1994;8:345-350.

70. Friis-Moller N, Thiebaut R, Reiss P, Weber R, Monforte AD, De Wit S, El-Sadr W, Fontas E, Worm S, Kirk O, Phillips A, Sabin CA, Lundgren JD, Law MG and group DADs. Predicting the risk of cardiovascular disease in HIV-infected patients: the data collection on adverse effects of anti-HIV drugs study. *Eur J Cardiovasc Prev Rehabil*. 2010;17:491-501.

71. Data Collection on Adverse Events of Anti HIVDSG, Weber R, Sabin C, Reiss P, de Wit S, Worm SW, Law M, Dabis F, D'Arminio Monforte A, Fontas E, El-Sadr W, Kirk O, Rickenbach M, Phillips A, Ledergerber B and Lundgren J. HBV or HCV coinfections and risk of myocardial infarction in HIV-infected individuals: the D:A:D Cohort Study. *Antivir Ther*. 2010;15:1077-86.

72. Bedimo R, Westfall AO, Mugavero M, Drechsler H, Khanna N and Saag M. Hepatitis C virus coinfection and the risk of cardiovascular disease among HIV-infected patients. *HIV Med*. 2010;11:462-8.

73. Lang S, Mary-Krause M, Simon A, Partisani M, Gilquin J, Cotte L, Boccara F, Costagliola D and French Hospital Database on HAC. HIV replication and immune status are independent predictors of the risk of myocardial infarction in HIV-infected individuals. *Clin Infect Dis*. 2012;55:600-7.

74. Mary-Krause M, Cotte L, Simon A, Partisani M, Costagliola D and Clinical Epidemiology Group from the French Hospital D. Increased risk of myocardial infarction with duration of protease inhibitor therapy in HIV-infected men. *AIDS*. 2003;17:2479-86.

75. Holmberg SD, Moorman AC, Williamson JM, Tong TC, Ward DJ, Wood KC, Greenberg AE and Janssen RS. Protease inhibitors and cardiovascular outcomes in patients with HIV-1. *The Lancet*. 2002;360:1747-1748.

76. Armah KA, Chang CC, Baker JV, Ramachandran VS, Budoff MJ, Crane HM, Gibert CL, Goetz MB, Leaf DA, McGinnis KA, Oursler KK, Rimland D, Rodriguez-Barradas MC, Sico JJ, Warner AL, Hsue PY, Kuller LH, Justice AC, Freiberg MS and Veterans Aging Cohort Study Project T. Prehypertension, hypertension, and the risk of acute myocardial infarction in HIV-infected and -uninfected veterans. *Clin Infect Dis*. 2014;58:121-9.

77. Choi AI, Vittinghoff E, Deeks SG, Weekley CC, Li Y and Shlipak MG. Cardiovascular risks associated with abacavir and tenofovir exposure in HIV-infected persons. *AIDS*. 2011;25:1289-98.

78. Oberai PC, Dalal D, Zhang L, Wang C, Eustace J and Parekh RS. Incidence of atherosclerotic cardiovascular disease among HIV patients receiving dialysis. *Am J Kidney Dis*. 2006;47:848-55.

79. Woolley IJ, Li X, Jacobson LP, Palella FJ and Ostergaard L. Macrolide use and the risk of vascular disease in HIV-infected men in the Multicenter AIDS Cohort Study. *Sexual health*. 2007;4:111-9.

80. Wever-Pinzon O, Bangalore S, Romero J, Silva Enciso J and Chaudhry FA. Inotropic contractile reserve can risk-stratify patients with HIV cardiomyopathy: a dobutamine stress echocardiography study. *JACC Cardiovasc Imaging*. 2011;4:1231-8.
